# Steps Toward Vitrification and Banking of Coral Larvae Using Field‐Friendly Cryomesh Technology

**DOI:** 10.1002/advs.78550

**Published:** 2026-11-10

**Authors:** E. Michael Henley, Jonathan Daly, Claire Lager, Nikolas Zuchowicz, Riley Perry, Jaylyn Oka, Mariko Quinn, Zongqi Guo, Maria Victoria Grosso‐Becerra, Frania Valdez Felix, Anastazia T. Banaszak, Christian R.A. Lippens, Kristen L. Marhaver, Emily Jane Williams, Keri O'Neil, Mary Hagedorn

**Affiliations:** ^1^ Smithsonian's National Zoo and Conservation Biology Institute Washington DC USA; ^2^ Hawaiʻi Institute of Marine Biology Kāneʻohe HI USA; ^3^ Taronga Institute of Science and Learning Taronga Conservation Society Australia Sydney Australia; ^4^ University of New South Wales Sydney Australia; ^5^ Department of Mechanical Engineering University of Minnesota Minneapolis MN USA; ^6^ Department of Mechanical and Aerospace Engineering University of South Florida Tampa FL USA; ^7^ Department of Medical Engineering University of South Florida Tampa FL USA; ^8^ Reef Systems Academic Unit Universidad Nacional Autónoma De México Mexico; ^9^ CARMABI Foundation Willemstad Curaçao; ^10^ Coral Conservation and Research Center The Florida Aquarium Tampa FL USA

**Keywords:** biobank, coral larvae, cryomesh, cryopreservation, heat transfer, vitrification

## Abstract

Coral reefs are declining globally, and novel solutions, such as cryopreservation, can help secure coral biodiversity and genetic diversity. Factors that affect successful cryopreservation of coral larvae include cryoprotectant type and concentration, membrane permeability, larval developmental stage, lipid content, volume, and freezing and warming rates. This study examined conduction‐dominated “cryomesh” technology across a range of coral species, distinguishing vitrification on cryomesh, 24 h swimming survival, and settlement competence as separate measures of success. Using a previously successful cryoprotectant solution, the exposure time, volume, and developmental stage most amenable to cryomesh cryopreservation were examined, and biophysical attributes were modeled to determine cooling and rewarming rates. Most species vitrified on the cryomesh and showed short‐term motility, but robust 24 h swimming survival was limited to *Lobactis scutaria* and *Fungia fungites*, the smallest species; other small‐volume species did not survive. To improve heat transfer, subsets of embryos were sheared during early development, yielding sub‐size larvae that did not swim post‐thaw, but the sheared, untreated controls settled and matured normally. These findings show that volume alone does not ensure survival. Additional next steps must include toxicity and permeation studies of other cryoprotectants and long‐term restoration potential evaluated by examining post‐thaw settlement success.

## Graphical abstract

Cryomesh enables a low‐tech cryopreservation method, adding a valuable option to the coral reef conservation toolkit. The approach reveals how size, toxicity, and developmental stage govern larval survival, while modeling links cooling and rewarming rates to a theoretical upper volume limit. Shearing embryos reduces volume below this bound, offering a promising strategy for corals with larger‐sized larvae.

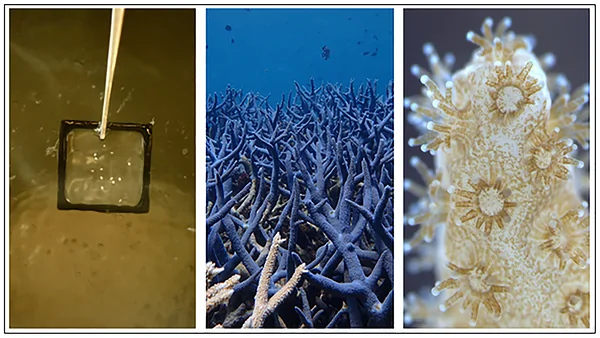

## 1 Introduction

Coral reefs are some of the oldest, most diverse ecosystems on Earth and among the most valuable. They play a critical role in maintaining ocean health, nurture more than 25% of all ocean life, protect homes and cities from storms, and add billions of dollars yearly to the global economy through fisheries, new pharmaceuticals, tourism, and recreation [1, 2, 3]. The world's coral reefs, however, continue to degrade at unprecedented rates [4, 5]. The overuse of fossil fuels is warming and acidifying the ocean [6, 7], producing a reef crisis that could lead to many coral species facing extinction within decades [8, 9]. Recent climate models estimate that if greenhouse gas emissions continue unabated, 95% or more of the world's shallow‐water reef‐building corals could die by the mid‐2030's [10]. Solutions are needed now to quickly ensure the integrity of global reef biodiversity and genetic diversity and to create robust genetic resources for future restoration.

Reducing global carbon emissions is essential for the survival of global coral reefs. However, the pace of climate change is outstripping the ability to save many coral species and habitats. Therefore, critical intervention methods are needed now to safeguard species and reefs, ensure opportunities for recovery, and sustain remaining biodiversity [11]. Novel solutions, such as cryopreservation, can help secure the biodiversity and genetic diversity of coral. Cryopreservation removes some cellular water via osmotic gradients and replaces it with cryoprotectant solutions. Then, the cells are cooled (either slowly or rapidly) to liquid nitrogen temperatures, where they can remain in stasis for decades. Cryoprotectants, such as dimethyl sulfoxide (DMSO), can permeate cell membranes to stabilize them during cooling and reduce lethal intracellular ice formation [12, 13, 14]. In addition to permeating cryoprotectants, non‐permeating cryoprotectants, such as trehalose, can pull water from cells osmotically, thereby concentrating permeating cryoprotectants within the cytoplasm and reducing the probability of intracellular ice formation [15, 16]. These processes depend strongly on the biophysical properties of the plasma membrane that govern the permeability rates of both water and cryoprotectants across the membrane, as well as on the overall volume and geometry of the cell or tissue [17, 18]. As the size of a cell or tissue increases, the surface‐to‐volume ratio decreases, and transport of water and cryoprotectants becomes increasingly limiting. Consequently, larger tissues and organisms are substantially more challenging to cryopreserve successfully because diffusion distances increase and the probability of damaging ice formation rises [19, 20]. Successfully done, however, cryopreservation can maintain cells, tissues, and whole organisms in stasis, frozen and alive, for decades.

To date, institutions around the world have successfully cryopreserved coral sperm [21, 22] and coral larvae [23, 24] and have helped create coral biorepositories in the US (USDA's Animal Germplasm Program and Smithsonian Institution), Australia (Taronga Conservation Society), Curaçao (CARMABI Foundation), and Mexico (Universidad Nacional Autónoma de México). Additionally, practitioners have used cryopreserved sperm to produce “Assisted Gene Flow corals,” i.e., corals with parents from two geographically distinct regions, for both the Caribbean and the Great Barrier Reef [25, 26].

Cryopreservation of coral sperm has been possible for over 15 years [27], and there are now thousands of cryopreserved samples from over 55 species of reef‐building coral stored in biorepositories around the world [28]. Despite the value of these samples as genetic resources, the use of frozen sperm to produce new corals is limited by the availability of fresh oocytes. This, in turn, reduces the opportunities for using cryopreserved sperm to help diversify populations as natural populations are diminished. However, the ability to cryopreserve coral larvae is a crucial step toward offsetting the extinction threat to coral. Moreover, as banked larval resources increase, these frozen assets can produce coral recruits in the absence of reproductively active adult corals, thus enabling the deployment of new corals outside of limited spawning periods to reduce overall restoration costs long‐term.

Vitrification and laser‐warming experiments have demonstrated that cryopreservation of coral larvae is possible, with the successful recovery of live larvae from the gonochoric spawning coral *Lobactis scutaria* [23] and the brooding coral *Stylophora pistillata* [29]. While laser nano‐warming is a suitable proof‐of‐concept, this approach utilizes expensive equipment requiring specialist training, requires rigorous permitting in laboratories, and relies on manual handling of few larvae in small sample volumes. These factors limit both accessibility and sample throughput, making this technology difficult to scale up to the numbers required to support banking and restoration.

As the extinction threat to coral has become alarming, especially in less diverse ecosystems such as the Caribbean, small‐scale larval vitrification cannot keep pace with conservation needs. Therefore, there is a need to create more field‐hardy, scalable, and easily transferable methods to vitrify coral larvae. Vitrification uses a cocktail of concentrated cryoprotectants that causes the sample to form a glass, preventing lethal intracellular ice formation. Cryomesh technology, a method that uses metal mesh to rapidly conduct heat away from cells, has been used to vitrify *Drosophila* embryos [30], bovine oocytes [31], plant shoots [32], and coral larvae [24], generally with good post‐thaw survival at approximately 50–80%. Cryomesh technology can achieve extremely high cooling rates because the sample preparation process removes excess cryoprotectant from around the samples through the mesh pores, thereby minimizing the total thermal mass. A recent study evaluated the basic heat transfer principles behind the cryomesh approach and investigated critical design parameters to achieve a conduction‐dominated cryomesh [24]. The conduction‐dominated cryomesh dramatically increases the cooling rate from 2‐ to 10‐fold, resulting in a cooling rate of 0.24 to 1.2 × 10^5^°C/min in various biosystems. The rate of freezing and warming using cryomesh is related to both the volume of the sample [24, 30] and the cryoprotectant permeability.

Our objective in this study was to apply conduction‐dominated cryomesh technology to larvae from a wide range of coral species to provide an initial guide for coral cryopreservation at this life stage. Because the volume of the larvae is a critical factor in determining whether the cryomesh can warm them rapidly enough, we started by measuring egg and larval volumes of a variety of Pacific and Caribbean coral species to determine whether there is a specific cutoff size for cryomesh cryopreservation. We developed a model of the ideal suite of constrained parameters that may lead to success for a particular coral species. In addition, we tested the toxicity of the vitrification solution (used in [23]) at different larval ages, conducted vitrification tests, and banked those species that survived the vitrification process. To build this framework, we examined a wide variety of larvae from the following species: *Lobactis scutaria* (Hawaiʻi), *Pocillopora meandrina* (Hawaiʻi), *Pocillopora acuta* (Hawaiʻi), *Fungia fungites* (Australia), *Acropora spathulata* (Australia), *Dendrogyra cylindrus* (Florida), *Mussa angulosa* (Florida), *Colpophyllia natans* (Florida), *Pseudodiploria strigosa* (Florida), *Diploria labyrinthiformis* (Mexico), *Orbicella annularis* (Mexico), and *Orbicella faveolata* (Curaçao and Mexico). See Figure 1 for a summary of larval volumes for these species. Next, we used these data to build a predictive model of how larval volume impacts freezing and warming rates in the conduction‐dominated cryomesh cryosystem.

**FIGURE 1:**
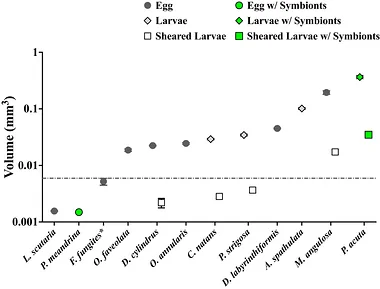
Parameters that influence successful preservation in coral. Volume represented logarithmically for various eggs and larvae for a variety of species. The dotted line represents the theoretical volume that can be successfully vitrified and warmed with cryomesh technology (∼0.005980 mm^3^). Five species were sheared early in development to produce smaller‐sized larvae, and three were under the targeted theoretical volume. The remaining large‐sized larvae were not sheared. An additional parameter to consider is the presence or absence of algal symbionts , which require additional steps. *Dendrogyra cylindrus*, *C. natans*, *P. strigosa*, *M. angulosa*, and *P. acuta* have two volume data points – a whole larvae or egg volume and a sheared larvae volume. *Fungia fungites larvae were successfully vitrified, but the volume measurements used here are derived from measurements found in Baird [42].

## 2 Methods

### 2.1 Coral Husbandry, Breeding, and Health

#### 2.1.1 Hawaiʻi

Colonies of *L. scutaria* and *P. meandrina* were collected from patch reefs in Kāneʻohe Bay, Hawaiʻi under permit SAP 2024–45. Hawaiian species were kept in outdoor aquarium tanks with flow‐through seawater until they were ready to be used for spawning. Spawning events occurred around the full moon in July and September 2024 (*L. scutaria*); May 2025 (*P. meandrina*); July and August 2025 (*L. scutaria*). During spawning, *L. scutaria* individuals were transferred to bowls filled with 0.22 µm filtered seawater (FSW). Individuals were allowed to spawn either eggs or sperm into their respective bowls. Before fertilization occurred, eggs were collected from each individual female for volume analysis. Eggs were then fertilized with pooled sperm and left undisturbed for 1 h. Bowls were moved into a covered area at ∼26°C, and gentle water changes were performed to remove used sperm. Larvae were cleaned each morning with FSW before the start of experiments. For *P. meandrina*, two to three colonies (genotypes) were placed in separate bins and were allowed to spawn and fertilize in the same tanks. From these, pools of larvae (n = 12, where n is a cross between 2–3 colonies) were then reared in separate bowls as above but with LED lighting to ensure the symbionts in the larvae were active.

#### 2.1.2 Australia

*Fungia fungites*, maintained long‐term at the Australian Institute of Marine Science (AIMS) National Sea Simulator facility (SeaSim), were isolated in three‐liter bowls prior to sunset, and they spawned at 7 PM. Their eggs and sperm were crossed as described for *L. scutaria* with seven sperm and egg donors (n = 3 males and n = 4 females). Afterward, the larvae were pooled and placed into a single 80‐L flow‐through rearing system with gentle flow and aeration using 0.2‐µm‐filtered seawater and a 60‐µm banjo filter on the outflow. *Acropora spathulata* was collected from Davies Reef under Great Barrier Reef Marine Park Authority permit #G23/ 49085.1 and with the free, prior, and informed consent of the Bindal Sea Country Traditional Use of Marine Resources Agreement committee. Colonies were collected in the week prior to coral spawning by AIMS divers and brought to the SeaSim facility at AIMS in Townsville, where they were maintained in flow‐through aquaria with shaded natural light at temperature profiles matching their natal reefs (∼27°C).

#### 2.1.3 Florida

Colonies of *D. cylindrus* were collected in Florida under Florida Keys National Marine Sanctuary permit FKNMS‐2015‐156. After collection, corals were transferred and loaned to the Florida Aquarium for long‐term holding. The colonies were held ex situ for several years under natural lighting conditions in greenhouses with recirculating tanks. Colonies were moved to an indoor spawning lab with LED lighting in the spring of 2024 and exposed to seasonal cues following procedures described in O'Neil et al. [33]. Spawn was collected and larvae produced in August 2024. Colonies were allowed to spawn in their respective tanks, and eggs and sperm were mixed in the water column and allowed to sit for approximately 20 min post‐spawn. Presumably fertilized eggs were then gently scooped or pipetted out of the tank into containers of filtered seawater for development. Once the embryos were at the 2‐cell stage of division, a subset of the dividing embryos was intentionally sheared to create smaller larvae. The cells were sheared apart by pouring the embryos from one bowl to another from approximately 1 m height. After shearing, all embryos were left to develop at ∼26.5°C until gastrulation. After gastrulation, larvae were cleaned each day by removing lipids from the surface of the water with plastic cling film and by picking out healthy larvae from the rearing containers with pipettes to move them to clean containers with filtered seawater. The sheared embryos were not suitable for experimentation for several days as they had membrane blebbing and irregular shapes. However, after about two days of healing time, when the larvae were 3 days (72 h) old, they exhibited normal larval morphology, swimming, and robustness.

Colonies of *Mussa angulosa* were collected under FKNMS permit FKNMS‐2017‐100 and/or FKNMS #2018‐139. After collection, corals were transferred and loaned to The Florida Aquarium for long‐term holding. The colonies were held ex situ for several years in recirculating tanks under natural lighting conditions in greenhouses. Spawn was collected and larvae produced in August 2024. The process from spawning to larval cleaning after gastrulation was the same as for *D. cylindrus*.

Colonies of *Colpophyllia natans* and *Pseudodiploria strigosa* were collected under FKNMS permit FKNMS‐2017‐100 and/or FKNMS #2018‐139. After collection, corals were transferred and loaned to The Florida Aquarium for long‐term holding. The colonies were held ex situ for several years under natural lighting conditions in greenhouses with recirculating tanks. For both *C. natans* and *P. strigosa*, colonies were moved to an indoor spawning lab with LED lighting in the fall of 2024, and spawn was collected and larvae produced in the fall of 2025. Colonies were allowed to spawn in their respective tanks, and the bundles of eggs and sperm were scooped out of the tank. Batch crosses were made by combining bundles from several colonies and allowing them to fertilize for approximately 30 min. After fertilization, the embryos were moved into rearing containers for development. Once the embryos were at the 2‐cell stage of division, a subset of the dividing embryos was sheared with gentle shaking on an orbital shaker at 58 RPM for 8 min. Sheared embryos were left to divide again into the 2‐cell stage, and the shearing process was repeated at 58 RPM for 12 min to create larvae at the equivalent of the 4‐cell division stage. After shearing, the process for larval development and cleaning was the same as for *D. cylindrus*.

#### 2.1.4 Curaçao

Larvae of the mountainous star coral *Orbicella faveolata* (formerly *Montastraea faveolata*) were reared from wild‐caught gamete bundles following annual mass spawning events. Divers monitored colonies from nights 5 to 8 after the September and October full moons from approximately 2h45m to 4h15m after sunset. Gamete bundles were collected using weighted polyester nets affixed to funnels and 50 mL conical tubes (BD Falcon). Gamete bundles were transferred to the laboratory and mixed in a 1L fat separator (Norpro), left to fertilize for 60 min, then rinsed 5 to 7 times with filtered seawater until the water ran clear. Filtered seawater was prepared from raw ocean seawater using sequential 10″ filter cartridges in polycarbonate housings with pore sizes 50, 20, 5, and 0.5 µm (H2O Distributors). Aliquots of the rinsed, fertilized eggs were transferred, using sterile Petri dish lids as scoops, into clear polystyrene clamshell deli containers (Dart Container Corporation), each containing approximately 800 mL of filtered seawater, and left to incubate overnight at ambient seawater temperature (27°C–28°C). Dead larvae and debris were removed by blotting with a laboratory wipe (Kimwipe) and by selective pipetting. Water changes were conducted every 1–2 days by transferring larvae by pipette into new containers. Collection and propagation work was conducted under the permits granted to CARMABI Foundation by the Government of Curaçao (Permit #2012/48584).

#### 2.1.5 Mexico

Prior to the 2024 and 2025 spawning seasons, monitoring of sites and colonies suitable for gamete collection was conducted in the Puerto Morelos Reef National Park (PNAPM). Subsequently, and in accordance with the spawning prediction calendar for this region of the Caribbean, gametes of *Diploria labyrinthiformis*, *Orbicella faveolata*, and *Orbicella annularis* were collected as authorized under permits issued by the Dirección General de Ordenamiento Pesquero y Acuícola No. PPF/DGOPA‐070/20 and No. PPF/DGOPA‐051/25 for *D. labyrinthiformis*, and by the Dirección General de Vida Silvestre of SEMARNAT No. SPARN/DGVS/09459/24 and SBRA/DGVS/04385/25 for the *Orbicella* species protected under Mexican law (NOM‐059). Gamete collection was performed by SCUBA diving at night, using conical nets positioned vertically over the colonies a few minutes before gamete release. The gametes were concentrated in the collection tube located at the top of the net. After spawning finished, the tubes were collected, capped, and maintained during transfer to the laboratory for assisted fertilization under controlled temperature conditions (27°C ± 1°C) using seawater filtered to 1 µm and sterilized with UV‐C light (FSW).

In August 2024, gametes from three *D. labyrinthiformis* colonies were combined in equal proportions, whereas in September, gametes from two *O. faveolata* colonies were used. Once fertilization was confirmed, excess sperm was removed by three consecutive FSW washes of the embryos at 28°C. Daily 70% water exchanges were performed until the larval stage was reached. Larvae were maintained in FSW with daily exchanges throughout the rearing period. Four‐ and five‐day‐old *D. labyrinthiformis* larvae and two‐ and three‐day‐old *O. faveolata* larvae were used for measurements.

In July and August 2025, gametes from two and three colonies of *D. labyrinthiformis*, respectively, were pooled for assisted fertilization. In September 2025, gametes from four colonies of *O. annularis* were used for assisted fertilization and combined in equal proportions. Embryo and larval care were carried out following the procedures previously described. For larval measurement assays, *O. annularis* larvae aged 8 and 9 days were employed.

### 2.2 Volume Measurements for Coral Eggs and Larvae

Precise volume measurement is needed to inform successful cryopreservation. Eggs and larvae were imaged in different locations using different compound microscopes. The compound microscope used for imaging *L. scutaria*, *P. meandrina*, *P. acuta*, *D. cylindrus*, *M. angulosa*, *C. natans*, *D. labyrinthiformis, O. annularis, O. faveolata*, and *P. strigosa* was an Olympus CX43 (Model CX43RF) with a trinocular port and digital microscope camera (AmScope MU2003‐BI) or an Olympus digital camera (Model EP50). A Leica Orthoplan (model 957685) with a Canon EOSR7 was used to measure *O. faveolata*, and *A. spathulata* was imaged with an Olympus BH‐2 with a Science Supply View camera (model S01‐0801A).

The larvae were pipetted into 1‐µl wells made from a standard glass microscope slide covered with a double thickness of standard electrical tape (3 M Scotch Electrical tape). A 2‐mm biopsy punch was used to create a small flat well in the electrical tape. A single egg or larva was transferred in 0.4–1 µl of FSW using a 2‐µl pipette with a cut tip and placed into the well. A pipette tip was used to flatten the water level in the well to reduce any possible magnification error from a concave or convex water surface. The eggs or larvae were imaged using a compound microscope at a magnification power that produced the largest image possible while still being in focus, usually a 10–40× objective. Images were captured with ToupLite software (https://sios.net.au/software/touplite) or EPview 1.4 for the Olympus EP50 camera (https://evidentscientific.com/en/downloads). A micrometer (OMAX Model A36CALM1 or Euromex Model AE.1110) was included when the egg/larval images were taken to calibrate dimensions of the sample later during analysis. Eggs or larvae from each genotype or pool were collected and imaged on developmental days ranging from D0 (eggs, day of coral spawning) to D9, depending on species. Once all images were taken, volumes were calculated by measuring the eggs and larvae using ImageJ software (ImageJ 1.54 g http://imagej.org). The shape of the eggs and larvae approximated an oblate spheroid; therefore, an ellipse was fitted to the image to acquire the semi‐minor and semi‐major axes of the planar image. Then a volume for each image was calculated using the equation for an oblate spheroid, V = 4/3 ´ π ´ (b)^2^´ c (b = semi‐major axis, c = semi‐minor axis).

#### 2.2.1 Shearing Larvae to Reduce Volume

Some of our early success with the cryomesh vitrification of coral larvae [24] was not readily transferable to species with larvae that had larger volumes, such as *Pocillopora acuta* and *Diploria labyrinthiformis* (data not shown). In August 2024, we held a workshop at The Florida Aquarium to test and standardize our cryomesh vitrification methods for coral larvae. It quickly became clear that the larger larvae were not amenable to vitrification; thus, we sheared subsequent cohorts of coral embryos during the early cell division stages to produce larvae with reduced overall volumes [34]. We applied this method to *D. cylindrus* embryos (prior to its listing as endangered under the U.S. Endangered Species Act) by shaking containers of larvae at the 2–8 cell stage or by pouring them from a height of approximately 1 m. Sheared *D. cylindrus* and *C. natans* larvae were settled and reared until maturity at The Florida Aquarium. These juvenile corals were held in recirculating tanks under natural lighting in greenhouses after settlement and treated with the same husbandry practices as all other recruits for the duration of their growth.

At a subsequent workshop at AIMS in 2025, embryos were dissociated using a combination of gentle shaking with an orbital shaker and pouring between two containers. Embryos at the 2‐cell stage were placed into square 2 L plastic containers on an orbital shaker and agitated at 59 RPM for 10 min, followed by pouring between containers from a height of approximately 20 cm. The dissociated embryos were allowed to divide further and were then dissociated again at the 2‐cell stage. At this point, blastomeres were equivalent in size to those of a normal 4‐cell stage embryo. This process was repeated one more time (equivalent to shearing embryos at the 8‐cell stage).

### 2.3 Modeling Vitrification and Rewarming Success Based on Volumes

A 1D numerical heat transfer model was developed in Excel to estimate achievable rewarming rates during vitrification‐based cryopreservation of coral larvae. The model incorporated conductive thermal resistances between the larvae, supporting substrate, and surrounding environment to simulate transient heat transfer during warming. Larval volumes used in the simulations were obtained from experimentally measured morphological data for each species and developmental stage. Using these experimentally derived geometries, the model was used to predict theoretical cooling and rewarming rates under different external conditions and evaluate whether the estimated critical cooling and warming threshold for suppressing devitrification could be achieved.

### 2.4 Symbiont Reduction and Feeding Coral Larvae

Some coral species transfer symbionts from the parent to the egg (*P. meandrina*) or larvae (*P. acuta*) during maturation [35]. However, the presence of photosynthetic symbionts (Symbiodiniaceae spp.) in the tissue causes complications during vitrification because the symbionts have different water and cryoprotectant permeabilities than the coral tissue [36, 37]. Because of this, larval symbiont ‘bleaching’ protocols were created to preserve the health of the larvae during vitrification (methods in [38]), with the understanding that coral symbionts could then be re‐introduced later after thawing. Briefly, the larvae were bleached using a menthol solution (99%, Sigma–Aldrich, St. Louis, MO) suspended in ethanol (190 proof, Decon Labs, Inc., King of Prussia, PA) paired with a constant 24‐h light period (no dark cycle) under fluorescent lights (∼350 PAR) at 26°C. A 200 mg/mL stock solution of menthol in ethanol was prepared (2 g menthol in 10 mL ethanol) and stored at 4°C until use. Larvae were placed in 2‐L aquaria, and 500 µl of the stock solution was added per 1 L of FSW (final concentration 0.1 mg/mL) with aeration for approximately 5 h at 26°C. After 5 h, they were transferred to a new tank with fresh FSW and aeration and maintained under the same lights. This was repeated until the larvae were visibly bleached, usually 2–3 days. Once the larvae were visibly bleached, they were returned to a 12 h day/night cycle and fed daily with commercially available amino acid supplements (Aquaforest Amino Mix or Brightwell Aquatics CoralAmino), adding 15 drops/L for 15 min.

### 2.5 Toxicity and Vitrification Testing

In previous studies, Daly et al. [23] (using laser‐warming) and Guo et al. [24] (using conduction‐dominated cryomesh) both successfully cryopreserved and revived *L. scutaria* larvae with a vitrification solution that was comprised of 10% 1,2‐propanediol (Sigma–Aldrich, 398039), 5% dimethyl sulfoxide (DMSO; Sigma–Aldrich, D8418), and 1 (m) trehalose (Food‐grade trehalose dihydrate, Swanson, Catalog #SWU478) in 1X Phosphate‐Buffered Saline (PBS; Sigma Aldrich, P3813). Therefore, we tested the larval response of many coral species to this cryoprotectant mixture of propanediol, DMSO, and trehalose in phosphate buffer solution – hereafter “PDT” – to determine how long each could tolerate the solution without damage (e.g., disruption of membranes and/or swimming/survival rates). Some experiments were stand‐alone toxicity trials while others were paired with a vitrification trial. Toxicity and vitrification of *L. scutaria* larvae have been studied previously – they can withstand exposure to PDT for up to 120 s – therefore, the overarching goal for this species in these experiments was to determine the optimal equilibration and rehydration time, while for all other species, the main goal was to assess toxicity exposure limits and post‐thaw responses.

Larvae were pipetted onto a 316 stainless steel woven cryomesh (400 × 400 openings per inch, 0.035 mm aperture, Inoxia Brand, McMaster‐Carr Catalog #9319T189) that was attached to a 3D‐printed PLA frame [39], blotted on lab‐grade wipes to remove FSW, and placed into PDT for dehydration for the specified experimental time. Upon removal, the cryomesh was again blotted to remove PDT, followed by an equal rehydration time in 0.5 M trehalose in FSW, and then recovered in FSW and analyzed the next day. Dehydration and rehydration steps were performed in shallow Petri dishes by lowering the cryomesh onto an aliquot of the treatment solution. For recovery in FSW, each cryomesh was placed into a separate well of a polystyrene 6‐well cell culture plate. Care was taken to ensure that larvae on the cryomesh remained separate and did not overlap each other, which would potentially affect membrane exposure to cryoprotectant and rehydration solutions, and impede heat transfer in vitrification treatments. Dehydration and rehydration times tested were 30, 60, 90, and 120 s. This broad range of dehydration and rehydration times was chosen to help identify whether post‐thaw survival could be increased by either reducing cryoprotectant exposure time to minimize potential toxicity (e.g., 30 s), or by increasing exposure time to potentially increase dehydration and cryoprotectant permeation (e.g., 90 and 120 s), compared to the 60 s exposure time used in previous studies. Survival was determined by counting larvae at 24 h that were both swimming (moving) in a directional, progressive motion and morphologically similar to the control larvae; smaller and irregularly shaped fragments that were stationary or spinning in a circular motion were excluded. *Lobactis scutaria* larvae were tested for toxicity and vitrification at developmental days D3–D5, *P. meandrina* larvae were tested at D2–D4, *D. cylindrus* larvae were tested at D2–D3, and *F. fungites* larvae were tested on D5. *Diploria labyrinthiformis* larvae were assessed for toxicity and vitrification on D4, whereas sheared larvae were assessed on Days 4 and 5. *Orbicella annularis* larvae were tested between Days 6 and 9, and *O. faveolata* larvae on D5.

To test for successful larval vitrification and warming, cryomeshes loaded with larvae were 1) blotted on lab‐grade task wipes to remove FSW, 2) gently placed in PDT for dehydration with long forceps, 3) removed and blotted to remove excess PDT from the cryomesh and frame, 4) gripped with long forceps and vertically plunged into liquid nitrogen until vitrified (i.e., until LN2 stopped boiling), 5) quickly removed from liquid nitrogen and plunged vertically into a container of 0.5 (M) trehalose in FSW for warming, 6) transferred to a larger container of ambient temperature (∼26°C) 0.5 (M) trehalose in FSW, and 7) transferred to FSW overnight for recovery. Note that all toxicity exposure testing followed the steps above, omitting only the liquid nitrogen and warming steps. For full description of cryomesh construction and cryomesh handling, see the experimental section in Guo et al. [24].

Toxicity and vitrification tests with *Fungia fungites* were conducted early in the development of the cryomesh freezing method during a limited research trip, and there were very few larvae produced by this species during that cycle; therefore, less extensive testing was done on this species overall. However, like *L. scutaria*, in the laser warming process [23], the *F. fungites* larvae were exposed to three toxicity and vitrification trials with 60:60, 60:120, or 120:60 s dehydration‐to‐rehydration times. Approximately 100–200 larvae were loaded onto each cryomesh, placed in PDT, either exposed to liquid nitrogen (vitrified) or not (toxicity exposure), rehydrated in 0.5 m trehalose in FSW, and then transferred to fresh FSW. Experimental wells with larvae from all toxicity and vitrification trials were video recorded at 24 h post‐treatment. As above, the number of intact progressive swimming larvae was counted, excluding irregularly shaped fragments.

### 2.6 Testing Increased Warming Temperature

We noticed in July 2025 that cryopreserved, post‐thaw *L. scutaria* larval treatment replicates appeared to have more, smaller larvae than the untreated controls. We hypothesized that some of the larvae may have cracked during warming, with the resulting larval pieces surviving and continuing to develop. To test this, two cryomeshes, each with ∼200 D5 *L. scutaria* larvae, were vitrified on stainless steel cryomesh with PDT (60s dehydration and rehydration; see previous methods) and warmed at two temperatures, 26°C and 50°C in 0.5 (M) trehalose in FSW. The 26°C treatment was warmed and rehydrated in 0.5 (M) trehalose in FSW (26°C) as above. For the other treatment, the 0.5 (M) trehalose in FSW was heated to 50°C. The vitrified larvae on the cryomesh were quickly plunged into the 50°C trehalose for less than one second, and then immediately moved into ambient (26°C) trehalose for the remainder of the rehydration period. Volumes for most of the larvae in the two treatments above and the untreated controls were measured and their volume distributions plotted (26°C: n = 221; 50°C: n = 239; control D5 larvae: n = 99). In examining the videos from the *F. fungites* from 2022, they also appeared to show this same phenomenon of producing smaller, fragmented swimming larvae, but volume distribution analysis was not done on this cohort.

### 2.7 Banking Coral Larvae

Cryomeshes with approximately 200 vitrified larvae each were placed into a “cryobox”: a VWR CryoPro cardboard sample box (CAT#82007‐158) with a 3D‐printed insert designed to hold 80 cryomeshes [39] (Figure S1), which fits into standard rack systems in cryobanks. The first iteration of this cryobox used in Australia only held 10 cryomeshes (not shown), but the more recent version holds 80 cryomeshes (Figure S1). The 3D‐printed inserts were designed to hold the cryomeshes with the ability to maintain some liquid nitrogen within the cryobox to help maintain temperature during handling and transfer into a permanent bank.

The vitrification of samples and loading into the cryobox were done in a liquid nitrogen‐safe container so that both the cryomesh sample and the cryobox remained fully immersed in liquid nitrogen throughout handling. Cryomeshes were vitrified in a strict vertical position to minimize the Leidenfrost effect [40]. Once boiling stopped, the cryomesh was maintained continuously under liquid nitrogen and transferred into a slot in the 3D‐printed insert so that it was not exposed to the air at any point. When the cryobox was full, the VWR cardboard sample box lid was labeled, placed in liquid nitrogen until temperature equilibration, secured to the cryobox, and then rapidly moved into temporary liquid nitrogen storage until shipment to a biorepository could be arranged.

Cryomesh is designed to facilitate a high heat‐transfer rate, so it is very sensitive to temperature change, and therefore it needs to be maintained constantly at cryogenic temperatures (i.e., below –150°C). Even though a vapor‐phase liquid nitrogen dewar can be used for permanent storage, because of their temperature sensitivity, we strongly advise against it. If kept in vapor phase for storage, the cryomeshes must be moved to liquid phase nitrogen during handling and transfer of samples to minimize transient warming from the surroundings.

### 2.8 Statistical Analysis

#### 2.8.1 Volume

To examine changes in volume, we used the nonparametric Kruskal‐Wallis test with Dunn's post hoc multiple comparisons (separately per species) for *L. scutaria*, *P. meandrina*, *D. cylindrus*, *D. labyrinthiformis, O. annularis*, and sheared *C. natans* larvae. For larval volumes of *M. angulosa*, *C. natans*, *P. strigosa*, *O. faveolata*, and sheared *P. strigosa*, we used a one‐way ANOVA with Tukey's post hoc normality assumptions, and confirmed the results with Shapiro‐Wilk tests and diagnostic plots. Similarly, for both the sheared *D. cylindrus* and sheared *M. angulosa* larval volumes, ANOVA normality assumptions were verified with Welch's correction for unequal variances and Dunnett's T3 post hoc comparisons. Survival rates of settled normal‐size versus sheared larvae from both *D. cylindrus* and *C. natans* were analyzed with Fisher's Exact Test.

#### 2.8.2 Toxicity Exposure

Percent survival for *L. scutaria* and *D. cylindrus* toxicity was analyzed as a function of development day and exposure time using a beta‐binomial generalized model with a logit link (initial binomial models showed substantial overdispersion), with the response specified as the number of individual larvae that survived or died per trial. Interaction and main effects were analyzed using Type II Wald chi‐square tests with Tukey‐adjusted post hoc pairwise comparisons, and model assumptions for both were assessed and verified using simulation‐based residual diagnostics. Similarly, *P. meandrina* toxicity percent survival was also analyzed with a beta‐binomial generalized linear model (logit link) using the number of survived or died larvae per trial as the response, exposure time as a fixed effect, verification of model assumptions, and post hoc comparison. The *F. fungites* toxicity survival was analyzed with a one‐way ANOVA, but due to the low sample size, violation of assumptions could not be assessed and limits inferences.

#### 2.8.3 Cryopreservation Post‐Thaw Survival and Volume

Post‐thaw percent survival of cryopreserved *L. scutaria* larvae was analyzed as a function of two developmental days and three exposure times with a beta‐binomial generalized linear model with logit link, response as the number of larvae that survived versus died, and interaction, main effects, and post hoc tests as above. Additionally, percent survival of day five *L. scutaria* larvae (survived/died counts) was also analyzed with a beta‐binomial generalized linear model (logit link) using exposure time and warming temperature as predictors. As above, beta‐binomial models were selected due to overdispersion in the initial binomial models, and further model assumptions were verified with simulation‐based residual diagnostics and plots for both. The *F. fungites* post‐thaw survival was analyzed with a one‐way ANOVA, but as above, the low sample size limits model verification and interpretation (more on this below). Post‐thaw volumes for the D5 *L. scutaria* control larvae and 26°C and 50°C warmed larvae were analyzed with a Kruskal‐Wallis test with Dunn's post hoc multiple comparisons analysis.

GraphPad Prism software (version 11.0.2; San Diego, CA) was used for volume analyses, figures, and general descriptive statistics. Percent survival analyses for toxicity and cryopreservation were conducted in R (version 4.6.1; R Core Team, 2026) [41]. Beta‐binomial models were fit using the glmmTMB package (version 1.1.14). Model fit and residual diagnostics were assessed using the DHARMa package (version 0.5.0), including simulation‐based tests for overdispersion and outliers. Type II Wald chi‐square tests for fixed effects were conducted using the car package (version 3.1.5). Estimated marginal means and Tukey‐adjusted pairwise comparisons were obtained using the emmeans package (version 2.0.4), and compact letter displays summarizing groups of statistically indistinguishable levels were generated using the multcomp (version 1.4.31) and multcompView (version 0.1.12) packages.

## 3 Results

Cryomesh cryopreservation had been successfully demonstrated in coral larvae in only a single species [24]. We hypothesized that a key element of success is the amount of heat transfer possible using cryomesh, determined largely by the volume of the tissue, but certainly, the physiological response to the vitrification medium (toxicity) and ability to vitrify (no visible ice formation) on the mesh were important as well. In this study, we examined the larvae of 12 species. Here, we present data for four species with approximately the same larval volumes, each with different biological aspects and outcomes, to illustrate some of the important elements leading to successful cryomesh vitrification and post‐thaw survival. These species are: 1) two species of coral (*L. scutaria* and *F. fungites*) with aposymbiotic larvae (lacking photosynthetic symbionts) that successfully vitrified, warmed, and survived (i.e., progressive forward movement with intact morphology), 2) one coral species (*P. meandrina*) whose larvae have symbionts and which did vitrify but did not survive post‐thaw warming, and 3) one coral species (*D. cylindrus*) whose aposymbiotic larvae were sheared to a smaller size, below the target volume for vitrification, that also vitrified but did not successfully survive after warming. The remaining data for other species can be found in the Supporting Information files.

### 3.1 Volume Measurements of Eggs and Developing Larvae

It was important to measure the volume of the eggs and larvae at each developmental stage of interest in case there were large differences between the egg and later developmental stages. Therefore, we measured eggs and developing larvae in a number of species (see Table 1 and Table S1 for descriptive statistics). The smallest larvae measured were *L. scutaria* and *P. meandrina*, with volumes ranging from ∼0.002 to ∼0.003 mm^3^. *Fungia fungites* size was estimated with data from eggs measured by Baird [42] and had a similarly small volume (∼0.005 mm^3^).

**TABLE 1:** Volume [mm^3^] of coral eggs and larvae across developmental days. Eggs or larvae were imaged on the day of spawning (D0, eggs) through developmental Day 5 (D5), depending on species. The sheared *D. cylindrus* larvae were broken apart at the 2–8 cell stage the night of spawning, and their volume measurements began on D1. The sample size (n) refers to the number of eggs or larvae measured at each time point.

*L. scutaria*	Day 0	Day 1	Day 2	Day 3	Day 4	Day 5
Genotypes	13	n/a	n/a	10	12	10
n	160	n/a	n/a	120	139	99
Median [mm^3^]	0.0014	n/a	n/a	0.00184	0.0018	0.0021
Mean [mm^3^]	0.0015	n/a	n/a	0.0019	0.0019	0.0022
SD	0.0004	n/a	n/a	0.0008	0.0007	0.0008
SEM	0.00003	n/a	n/a	0.00008	0.00006	0.00008

*P. meandrina*	Day 0	Day 1	Day 2	Day 3	Day 4	Day 5
Genotypes	11	8	6	6	6	n/a
n	110	80	60	60	60	n/a
Median [mm^3^]	0.0014	0.0019	0.0026	0.0026	0.0039	n/a
Mean [mm^3^]	0.0015	0.0022	0.0032	0.0030	0.0041	n/a
SD	0.0002	0.0008	0.0017	0.0012	0.0016	n/a
SEM	0.00002	0.00009	0.0002	0.0001	0.0002	n/a

*D. cylindrus*	Day 0	Day 1	Day 2	Day 3	Day 4	Day 5
Genotypes	4	8	6	4	4	n/a
n	40	80	60	40	40	n/a
Median [mm^3^]	0.0220	0.0262	0.0226	0.0220	0.0176	n/a
Mean [mm^3^]	0.0224	0.0274	0.0237	0.0229	0.0192	n/a
SD	0.0029	0.0053	0.0043	0.0057	0.0083	n/a
SEM	0.0004	0.0006	0.0005	0.0009	0.0013	n/a

*D. cylindrus—Sheared*	Day 0	Day 1	Day 2	Day 3	Day 4	Day 5
Genotypes	n/a	8	6	4	4	4
n	n/a	10	10	10	10	10
Median [mm^3^]	n/a	0.0023	0.0044	0.0030	0.0075	0.0057
Mean [mm^3^]	n/a	0.0022	0.0049	0.0033	0.0079	0.0054
SD	n/a	0.0013	0.0008	0.0014	0.0031	0.0007
SEM	n/a	0.0004	0.0003	0.0004	0.00097	0.0002

The median volume for *L. scutaria* eggs (D0, n = 230) was 0.001473 mm^3^, and the larvae increased to 0.002136 mm^3^ by D5 (n = 99). A Kruskal‐Wallis test indicated there was a significant change in larval volume across developmental days (*L. scutaria*, n = 518, H_3_ = 65.14, *p* < 0.0001). *Pocillopora meandrina* eggs had a similar median volume of 0.0014 mm^3^ (D0, n = 110), while D4 larvae increased to 0.0039 mm^3^ (day 4, n = 60), and a Kruskal‐Wallis test detected a significant change from egg to larval volume across developmental days (n = 370, H_4_ = 202.1, *p* < 0.0001). In both *L. scutaria* and *P. meandrina*, the post hoc comparisons showed increasing median volumes as development continued throughout the week. See Table 1 and Figure 2 for *L. scutaria* and Table 1 and Figure 3 for *P. meandrina*.

**FIGURE 2:**
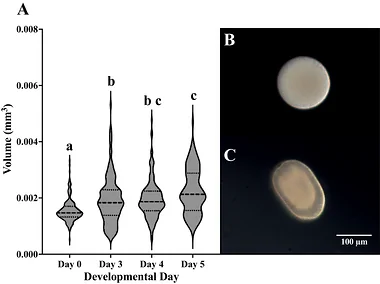
Volume comparison of *L. scutaria* across early development. A) Volume of eggs and developing *L. scutaria* larvae. B) Image of *L. scutaria* egg, and C) Day 4 *L. scutaria* larva. These data were collected from 21 genotypes from spawning seasons 2024 and 2025. At each developmental day, ten eggs or larvae from 13 genotypes were haphazardly selected and imaged from surviving genotypes. The dotted line is the median, and different letters above plots are statistically significant, indicating a slight increase in volume during development (n = 518, H_3_ = 65.14, *p* < 0.0001).

**FIGURE 3:**
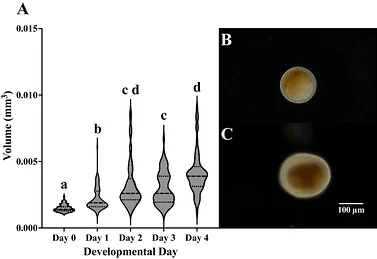
Volume comparison of *P. meandrina* across early development. A) Volume of eggs and developing *P. meandrina* larvae. B) Image of a *P. meandrina* egg and C) Image of a D4 *P. meandrina* larva. These data were collected from 11 pools consisting of 21 genotypes (two genotypes per pool). At each developmental day, ten eggs or larvae were imaged from each surviving pool, and there was a change in size during development (n = 370, H_4_ = 202.1, *p* < 0.0001). The dotted line is the median, and different letters above plots are statistically significant.

*D. cylindrus* eggs (D0, n = 40) were nearly an order of magnitude larger (∼0.022 mm^3^) than *L. scutaria*, *F. fungites*, and *P. meandrina* eggs. A subset of the developing *D. cylindrus* larvae at the 2‐ to 8‐cell stage were sheared to produce smaller‐sized, functional larvae. The median volume of *D. cylindrus* also changed significantly over the developmental days monitored, with the largest increase the day after spawning and fertilization (D1, n = 80; 0.0262 mm^3^), but declining significantly in volume (D4, n = 40; 0.0176 mm^3^) as development continued (n = 260, H_4_ = 55.6, *p* < 0.0001). See Table 1 and Figure 4A. Sheared D1 (n = 10) *D. cylindrus* larvae resulted initially in a median volume of 0.00233 mm^3^ that increased over time to 0.00568 mm^3^ on D5 (n = 50, W_4, 21.8_ = 14.99, *p* < 0.0001). See Table 1 and Figure 4B. Shearing reduced the mean volume of *D. cylindrus* by 68%. Specifically, the D4 median intact volume was 0.0176 mm^3^ vs. the D5 sheared volume of 0.00568 mm^3^.

**FIGURE 4:**
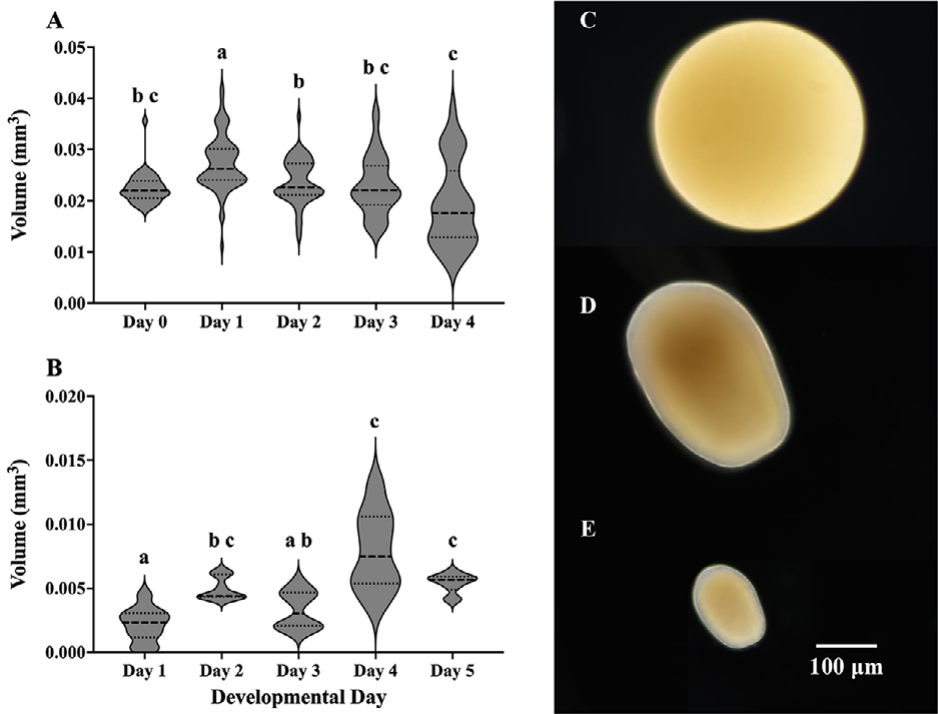
Volume comparison of *D. cylindrus* across early development, normal and sheared larvae. A) Volume of eggs and developing *D. cylindrus* larvae. These data were collected from 24 genotypes across 8 pools from spawning season 2024, and at each developmental day, 10 eggs or larvae from 8 genotypes were haphazardly selected and imaged from surviving genotypes. The dotted line is the median, and different letters above plots are statistically significant (n = 260, H_4_ = 55.6, *p* < 0.0001). B) Volume of developing *D. cylindrus* larvae that were sheared during the 2‐cell stage of development (approximately 3.5 h post‐fertilization). These data were collected from one pool of four genotypes, and at each developmental day, 10 sheared larvae were imaged from this pool. The large increase in volume at D4 may have been due to fusion of some sheared larvae. The dotted line is the median, and different letters above plots are statistically significant (n = 50, W_4, 21.8_ = 14.99, *p* < 0.0001). C) Image of a *D. cylindrus* egg. D) Image of a control (not sheared) D4 *D. cylindrus* larva. E) Image of a sheared D4 *D. cylindrus* larva.

An important question was whether these sheared coral larvae were viable and thereby useful for conservation since their reduced size and volume would potentially make them more amenable to the mesh cryopreservation system. To determine whether these sheared larvae would continue developing and become settlement competent and mature to normal‐sized coral, a small subset of the larvae were settled and held in human care (Figure 5).

**FIGURE 5:**
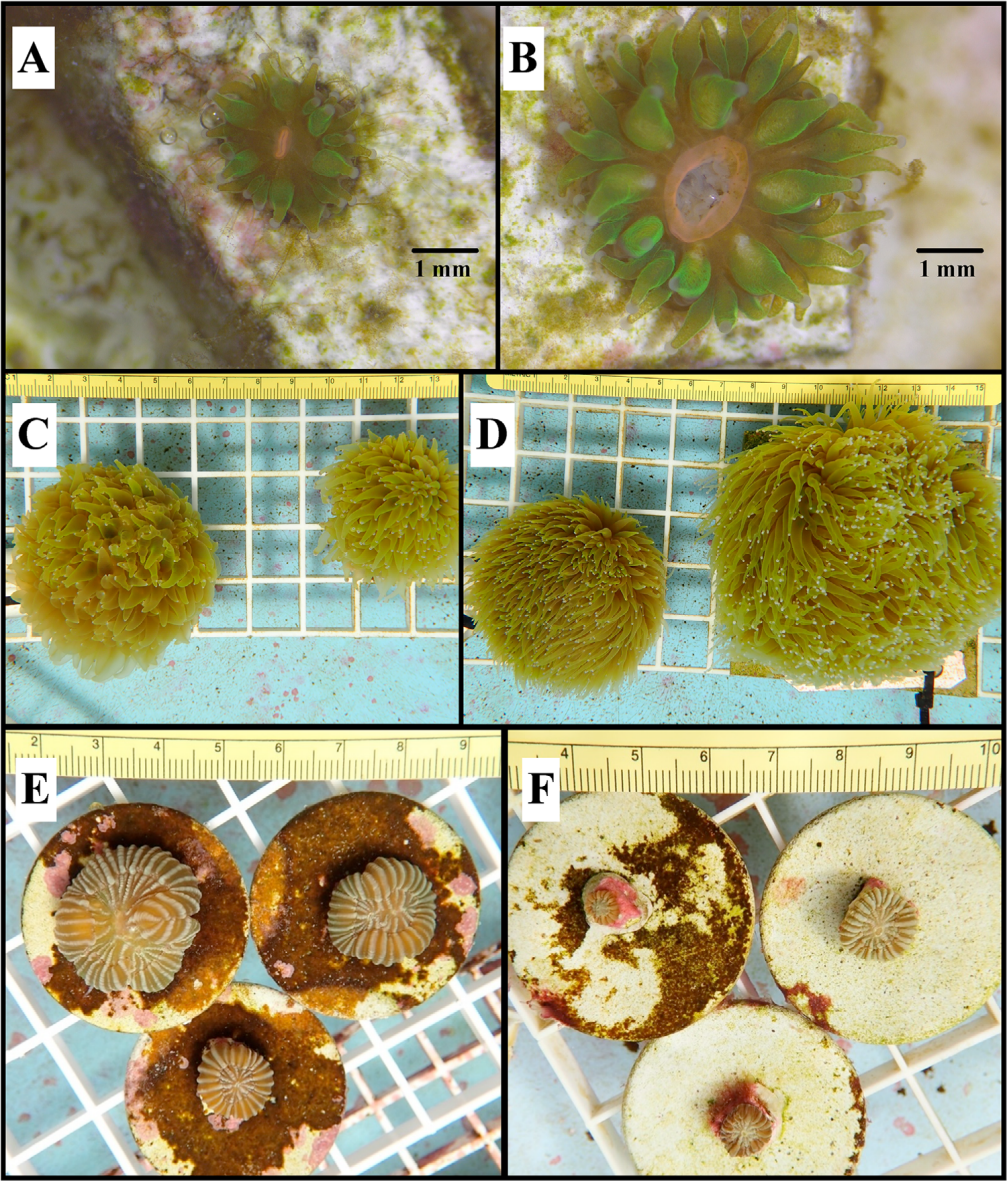
Growth and size of recruits from sheared larvae. A) *Dendrogyra cylindrus* recruits from an unsheared (control) larva at 5 months and B) from a sheared larva at 5 months. C) *D. cylindrus* recruits from unsheared larvae at 20 months and D) from sheared larvae at 20 months. E) *Colpophyllia natans* recruits from unsheared larvae at 8 months and F) from sheared *C. natans* larvae at 8 months old. Scale bars in panels A and B are 1 mm, and scale bars in panels C‐F are cm.

For *D. cylindrus*, the settlement and growth of the sheared larvae were comparable to unsheared larvae, and at 20 months, there was no difference in survival between the two groups of juveniles (Table 2, Fisher's Exact Test p = 0.4832). They showed no harmful side effects of the shearing process after settlement; in fact, the largest and fastest‐growing individual from this cohort was from the sheared group. *Colpophyllia natans* juveniles reared from sheared larvae mostly behaved similarly to those from the control (unsheared) larval cohort, but there was an observable difference in their growth. At eight months, the juveniles reared from sheared larvae were noticeably smaller than the control population but were otherwise visually healthy and continued to grow (Figure 5). Additionally, at eight months after settlement, there was no difference in survival between the two groups (Table 2, Fisher's Exact Test p = 0.4124). For the remaining coral species in this study, data on the larval volumes that were measured, described, and analyzed can be found in Table S1 and Figures S2–S7.

**TABLE 2:** Settlement and survival of sheared larvae. Larvae from *D. cylindrus* and *C. natans* settled and were raised at the Florida Aquarium Coral Conservation and Research Center. All corals were raised in the same conditions following the normal husbandry protocols of the facility. The survival for the sheared larvae is compared to the survival of unsheared larvae from the same cohort. There is no difference in survival of sheared vs. unsheared larvae for both *D. cylindrus* (Fisher's Exact Test p = 0.4832) and *C. natans* (Fisher's Exact Test p = 0.4124).

*D. cylindrus*	Initial number of recruits August 2024	Survival to May 2026 (20 months)	% Survival to May 2026 (20 months)
Sheared larvae	83	7	8.43%
Un‐sheared larvae	56	2	3.57%

*C. natans*	Initial number of recruits September 2025	Survival to May 2026 (8 months)	% Survival to May 2026 (8 months)
Sheared larvae	136	7	5.15%
Un‐sheared larvae	20 479	1577	7.70%

### 3.2 Cryoprotectant Toxicity Exposure

The cryopreservation solution (PDT) did not appear to have a large negative impact on *L. scutaria* larvae as percent survival was relatively consistent across the four developmental days and three exposure times tested. However, there were some interactions noted. Specifically, percent survival ranged from a mean of 68% (D4, 90 s) to 87% (D2, 120 s; see Table S2). The developmental day by exposure time interaction was not significant (n = 95 trials, χ^2^(6) = 4.30, p = 0.6359) and was removed to investigate main effects only. Within this model, developmental day was a significant factor (χ^2^(3) = 14.16, p = 0.0027), but exposure time was not (χ^2^(2) = 2.19, p = 0.334). Because developmental day was significant (but not exposure time), estimated marginal means for developmental day (averaged over exposure time) were compared using Tukey‐adjusted pairwise comparisons. The post hoc test determined that mean survival probability on developmental D4 (∼69%) was significantly lower than D2, D3, and D5 (∼78%, 82%, and 79%, respectively). See Figure 6.

**FIGURE 6:**
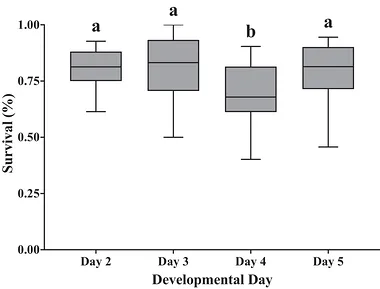
The effects of developmental day and vitrification solution exposure on *L. scutaria* larvae.​ Comparison of cryoprotectant exposure times and developmental day in *L. scutaria* larvae revealed that there was no interaction among the two (n = 95 trials, χ^2^(6) = 4.30, p = 0.6359). Development day significantly affected survival (p = 0.0027), while exposure time did not (p = 0.334). Survival at D4 was lower than at D2, D3, and D5, which did not differ. Plots are min to max, and the line is the median. Different letters above plots are statistically significant.

Similar to *L. scutaria*, for *P. meandrina* there was no overall toxicity noted from the cryoprotectant exposure, as no difference in percent survival between the two exposure times of 30 s (∼76%, n = 9 trials) and 60 s (∼67%, n = 12 trials) on D4 was detected (OR = 1.08, z = 0.16, p = 0.871). See Table S2 and Figure 7.

**FIGURE 7:**
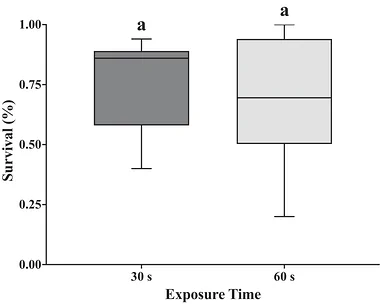
Toxicity exposure survival of *Pocillopora meandrina* larvae in PDT vitrification solution. Four‐day‐old *P. meandrina* larvae were exposed to PDT vitrification solution at two exposure times, and there was no difference in survival (OR = 1.08, p = 0.871). Plots are min to max, and the line is the median. Shared letters above plots indicate no statistical difference.

Survival of *D. cylindrus* larvae from exposure to the cryopreservation solution PDT was more variable but overall was still not significant. Survival of whole (intact) *D. cylindrus* larvae was examined with three exposure times over two developmental days, ranging from a mean percent survival of ∼35% (D3, 120 s, n = 4) to ∼62% (D2, 60 s, n = 4). See Table S2. Neither developmental day (χ^2^(1) = 2.35, p = 0.125), exposure time (χ^2^(2) = 1.12, p = 0.571), nor their interaction (n = 27 trials, χ^2^(2) = 0.01, p = 0.996) significantly affected percent survival (Figure 8). There were insufficient sheared *D. cylindrus* larvae to test toxicity.

**FIGURE 8:**
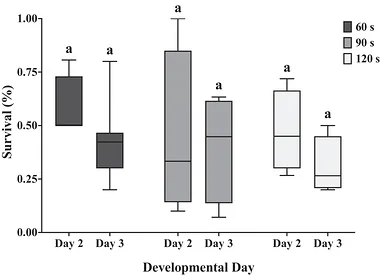
The effects of developmental day and vitrification solution exposure on *D. cylindrus* larvae. Neither exposure time nor developmental day significantly contributed to survival to PDT. There was no interaction between development and exposure time (n = 27 trials, χ^2^(2) = 0.01, p = 0.996). Additionally, there was no difference in survival based on different exposure times (p = 0.571) nor at different developmental days (p = 0.125). Plots are min to max, and the line is the median. Shared letters above plots indicate no statistical significance.

Finally, cryoprotectant exposure did appear to have some impact on the survival of *F. fungites* larvae. Specifically, the mean percent survival of D4 *F. fungites* larvae in PDT used three dehydration/rehydration treatment combinations (60 s:60 s = 34%; 120 s:60 s = 36%; 60 s:120 s = 54%). While a one‐way ANOVA did not find any difference among the three groups (n = 6 trials, F_2, 3_ = 3.221, p = 0.1791), model assumptions were not reliably verified due to the small number of total available larvae for experiments from this spawning event. These average values for percent survival were calculated with ∼100‐200 larvae per trial, but with only two trials for each treatment combination, the small number of trial sizes will limit reliable inferences (Table S2, Figure 9A).

**FIGURE 9:**
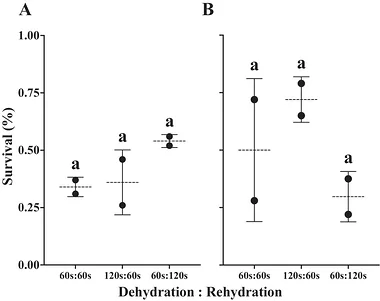
Survival of *F. fungites* from toxicity exposure and vitrification. A) Four‐day‐old *F. fungites* larvae were exposed to PDT vitrification solution at three exposure/rehydration intervals to assess toxicity. There was minimal difference in survival, but due to a limited number of larvae, only two trials were run for each treatment combination (each trial had 100–200 total larvae). The dark circles represent an individual percent survival per trial. The dotted line is at the mean, and error bars are SD. B) Survival of *F. fungites* after vitrification. Day 4 larvae were placed in PDT, vitrified on cryomesh, and rehydrated in 0.5 M trehalose. There was more variability in survival for the vitrified larvae than in the toxicity trials. However, as with toxicity, limited available larvae allowed for only two trials (n = 2) for each treatment combination (each trial had 100–200 total larvae). The dark circles represent the individual percent survival per trial. The dotted line is at the mean, and error bars are SD. Note: For both A) toxicity and B) vitrification, while each test did not detect significant differences (shared letters), the low number of available larvae only allowed for two trials per treatment for each, which limits the accuracy of the model and inferences.

### 3.3 Cryopreservation Post‐Thaw Survival and Volume

Both *L. scutaria* and *F. fungites* larvae survived vitrification, demonstrating normal, directional progressive movement and morphology at 24 h post‐thaw. Mean post‐thaw survival of D4 *F. fungites* larvae used three dehydration/rehydration combinations: 60 s:60 s, ∼50% survival; 120 s:60 s, ∼72% survival; and 60 s:120 s, ∼30% survival. A one‐way ANOVA did not find any significant difference among the three groups (n = 6 trials; F_2,3_ = 2.259, p = 0.2521), but, again, the number of available larvae for these experiments was very limited (Table S3 and Figure 9B). As with toxicity exposure for this species, there were only enough larvae for two trials with each treatment combination (∼100‐200 larvae per trial) to calculate post‐thaw percent survival. The small number of larvae that were available for this *F. fungites* cohort limited the number of trials needed to provide robust statistical analysis, yet those that were available survived the toxicity and cryopreservation process. Therefore, these data presented here should be considered primarily as an overview of a species that survived the entire process rather than the nuanced differences among dehydration and rehydration times. Additional testing is needed to optimize methods for a protocol.

Post‐thaw survival of vitrified *L. scutaria* larvae ranged from a mean of ∼46% (D4, 90 s) to ∼77% (D5, 90 s). A beta‐binomial model comparing two developmental days (D4 vs. D5) at three exposure times (60, 90, and 120 s) found that there was no interaction between developmental day and exposure time (n = 44 trials, χ^2^(2) = 0.82, p = 0.665). Exposure time (χ^2^(2) = 2.92, p = 0.233) and development day (χ^2^(1) = 0.85, p = 0.358) were also not significant. See Table S3 and Figure 10A.

**FIGURE 10:**
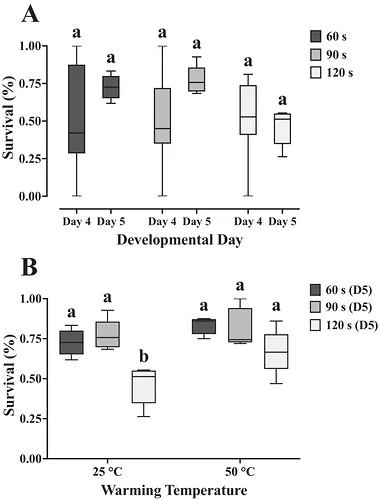
Influence of exposure time, developmental day, and warming temperature on the survival of *Lobactis scutaria* after vitrification. A) There was no difference in post‐thaw percent survival based on exposure time in PDT (p = 0.233) or developmental day (p = 0.358), nor was there a significant interaction between the two (n = 44 trials, χ^2^(2) = 0.82, p = 0.665). There is more variability in surviving D4 larvae (note that development day is near the α = 0.05 threshold). B) Increasing the warming temperature to 50°C on D5 larvae was only effective for the 120 s treatment. There was no interaction between exposure time and warming temperature (n = 34 trials, χ^2^(2) = 0.99, p = 0.609), but both exposure time (*p* < 0.0001) and temperature (p = 0.005) were significant. Plots are min to max, and the line is the median. Different letters above plots are statistically significant.

We also tested two different warming temperatures to thaw vitrified *L. scutaria* larvae, using those from developmental D5 only. Mean percent survival of thawed larvae ranged from a low of ∼49% (25°C, 120 s, n = 6) to a high of ∼85% (50°C, 60 s, n = 5). There was no interaction detected between warming temperature and exposure time (n = 34 trials, χ^2^(2) = 0.99, p = 0.609). Conversely, temperature (χ^2^(1) = 12.08, p = 0.005) and exposure time (χ^2^(2) = 33.91, *p* < 0.0001) were both significant factors; however, exposure time, rather than warming temperature, appears to have a stronger association with larval survival. See Table S3 and Figure 10B.

Increasing warming temperature to 50°C resulted in some beneficial post‐thaw survival characteristics, because it appeared to move the post‐thaw larval volume distribution more toward the volume distribution of the untreated control group. The Kruskal‐Wallis with post hoc test detected volume differences among all three groups (n = 559, H_3_ = 208.0, *p* < 0.0001). Specifically, the median volume differences among *L. scutaria* control and larvae warmed at these different temperatures were control larvae (0.002136 mm^3^, n = 99 larvae), 50°C larvae (0.001024 mm^3^, n = 239 larvae), and 26°C larvae (0.000671 mm^3^, n = 221 larvae). See Table S4 and Figure 11. These differences resulted in an approximately 52% and 69% decrease in median volume between the Control and 50°C warmed larvae and Control and 26°C warmed larvae, respectively.

**FIGURE 11:**
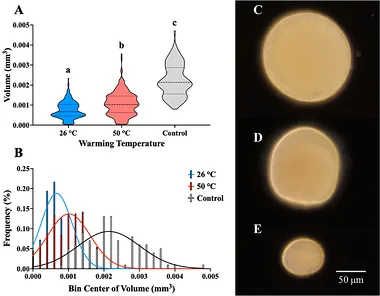
Comparison of *L. scutaria* post‐thaw larval volumes at two warming temperatures. A) Increasing the warming temperature from 26°C to 50°C also increased the median volume of the surviving larvae by nearly 20%, though volumes of both post‐thaw groups were still smaller than the control larvae (n = 559 larvae, H_3_ = 208.0, *p* < 0.0001). Dotted line is the median, and different letters above plots are statistically significant. B) Histogram distribution of post‐thaw *L. scutaria* larval volumes at two warming temperatures. Increasing the warming temperature of vitrified larvae from 26°C to 50°C shifted the post‐thaw volume toward the control (untreated) group, while also reducing the peak observed in the 26°C group. C) Control *L. scutaria* larva. D) Post‐thaw larva that was briefly warmed in 0.5 m trehalose at 50°C, then moved to 26°C 0.5 m trehalose. E) Post‐thaw *L. scutaria* larva that was warmed in 26°C 0.5 m trehalose. The surviving post‐thaw larvae maintained similar shape and swimming behavior to the control larvae, but they were reduced in size. The scale bar is 50 µm.

Cryopreservation trials with both *P. meandrina* and sheared *D. cylindrus* larvae on D4 and D3, respectively, were conducted. The larvae of both species demonstrated vitrification on the cryomesh (meaning no ice was visible by eye), and each species demonstrated limited movement in a handful of larvae at 2 h, but no consistent successful post‐thaw swimming at 24 h in either species was observed.

### 3.4 Modeling Vitrification and Rewarming Success Based on Volume

Based on the measured volume, we developed a thermal model to predict the cryomesh cooling in LN_2_ or rewarming in rewarming solution (Figure 12A, see details in Supporting Information). There are two processes of heat release: 1) convection heat transfer, which transfers the heat of the cryomesh and biosystem to liquid nitrogen (vitrification) or to the rewarming solution (rewarming), and 2) conduction heat transfer inside and between the cryomesh and biosystem. To describe the relative contributions to heat transfer, we analyzed a simplified thermal resistance model, which can be used to describe transient heat transfer in a model system. This model describes the heat flux (*q"*) during cooling, which will have a linear relation with the heat loss rate and thus cooling rate. We describe these conditions through a simple 1D thermal resistance model, which is

(1) $$q^{\prime \prime }=\frac{\Delta T}{R_{h}+R_{m}+R_{b}}=\frac{T_{i}-T_{\infty }}{R_{h}+R_{m}+R_{b}}=\frac{T_{i}-T_{\infty }}{R_{h,c}+R_{b,c}}$$

where *ΔT* is the temperature difference between the initial temperature of biosystem and final temperature (LN_2_ or rewarming solution), *R*_h_ is the external resistance of convection between the cryomesh and LN_2_, *R*_h,c_ is the external resistance of convection between the biosystem and LN_2_, *R*_m_ is the internal conduction thermal resistance of the cryomesh, and *R*_b_ and *R*_b,c_ are the conduction thermal resistances of the biosystem. The analysis primarily focused on rewarming rather than cooling because insufficient rewarming rates can lead to devitrification and intracellular ice formation during thawing, which is often one of the major limitations in vitrification‐based cryopreservation of larger and more complex biological systems [43]. The heat transfer analysis demonstrates that larval size is a dominant parameter governing achievable cooling and rewarming rates during vitrification‐based cryopreservation (Figure 12B and Figure S8).

**FIGURE 12:**
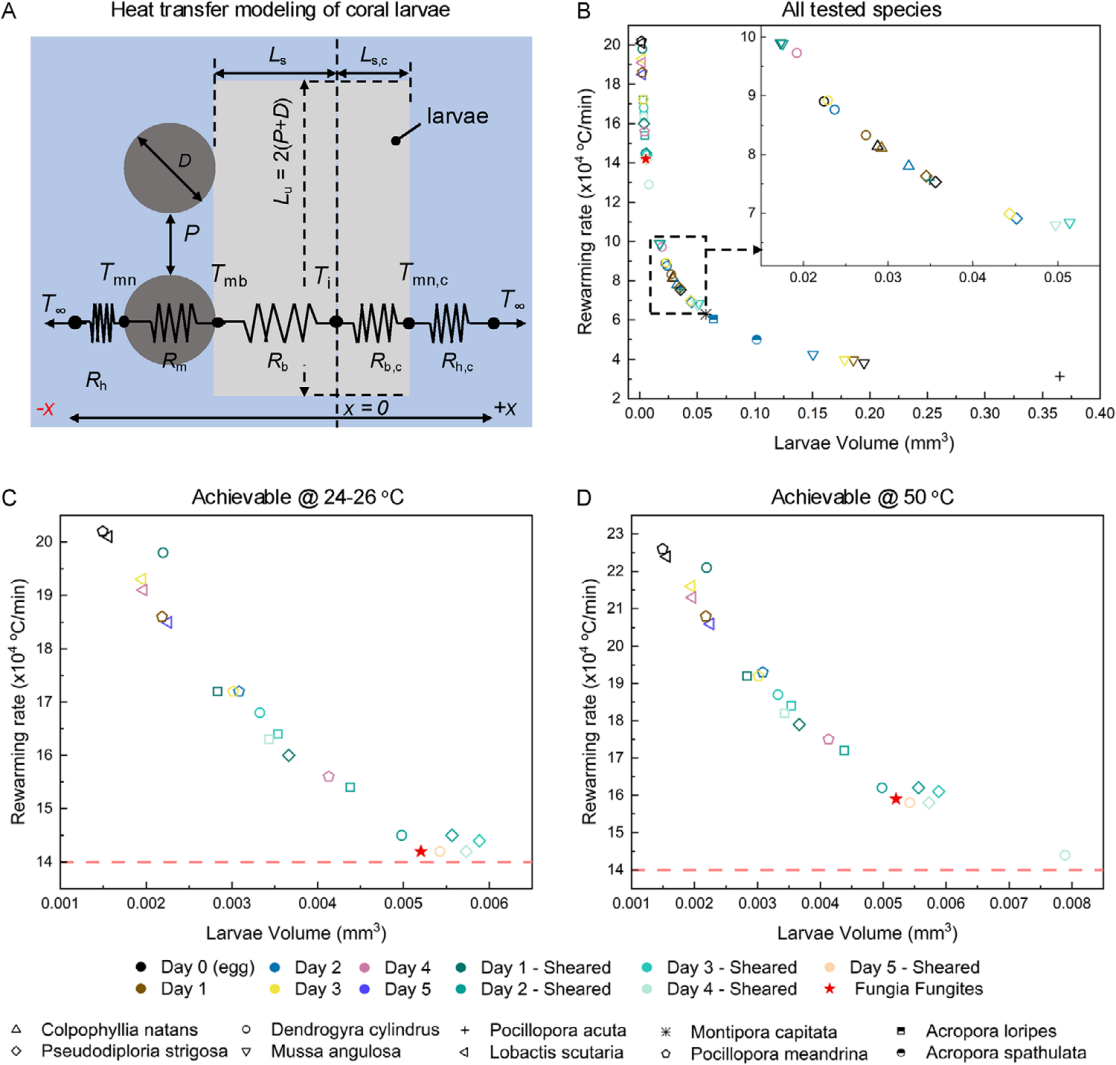
Heat transfer modeling and theoretical rewarming performance of coral larvae. A) Schematic of the heat transfer model used to estimate the rewarming and cooling rate of coral larvae on a cryomesh. B) Theoretical rewarming rate increases as larval volume decreases. C) Theoretical rewarming rates of rewarming solution at 24°C–26°C for coral larvae at different developmental stages and species. The dashed red line indicates the target critical rewarming rate required to minimize devitrification and ice formation during warming based on *F. fungites* cryopreservation experiments. D) Theoretical rewarming rates increase when the temperature of the rewarming solution increases to 50°C, showing possible improvement of viability due to a higher rewarming rate than at 24°C–26°C.

The previous successful cryopreservation of *F. fungites* is used as a threshold for cooling (7.5 × 10^4^°C/min) and rewarming rate (1.4 × 10^5^°C/min), shown as a red dashed line (Figure 12). Larval species and development stages with a theoretical rewarming rate exceeding the threshold can be rewarmed without devitrification. Across all tested coral species (Figure 12B,C), smaller larvae of *L. scutaria* (Figure S9A,B) and *P. meandrina* (Figure S9C,D) consistently exhibited substantially higher rewarming rates around 1.86 × 10^5^°C/min and 1.58 × 10^5^°C/min, respectively, as the lowest theoretical rewarming rate. In contrast, larger larvae showed a reduced rewarming rate compared with smaller larvae (Figure 12B), which had a rewarming rate below the threshold. Reduced rewarming rate is due to increased thermal mass and longer characteristic heat diffusion distances (i.e., *L*_s_ and *L*_s,c_), consistent with the thermal resistance‐based predictions of the heat transfer model.

The model also demonstrated that mechanically sheared larvae generally achieved higher theoretical rewarming rates than non‐sheared larvae at equivalent developmental stages, due to their reduced effective volume and thermal resistance (*R*_b_). Based on the heat transfer model, the sheared larvae with smaller volumes (*C. natans, D. cylindrus, and P. strigosa*) can achieve a rewarming rate that exceeds the threshold (Figure 12C and Figure S10). The theoretical rewarming rate of *D. cylindrus* at Day 1 increases from 8.41 × 10^4^°C/min to 1.47 × 10^5^°C/min at Day 1 – sheared, which is a 74% increase in rewarming rate and is beyond the threshold of the rewarming (Figure S10C). When the temperature of the rewarming solution increases from 24°C–26°C to 50°C, the theoretical rewarming rate is also increased (Figure 12D).

For larvae with a theoretical rewarming rate above the threshold, such as *L. scutaria* and *P. meandrina*, elevated warming temperatures may also reduce the required cryoprotective agent (CPA) concentration, thereby minimizing CPA‐associated toxicity while maintaining vitrification stability [44]. For species with a rewarming rate close to the threshold, increasing the temperature of the rewarming solution can raise it above the threshold. *D. cylindrus* at Day 4 sheared has an average rewarming rate of 1.28 × 10^5^°C/min at 24°C–26°C, while the average rewarming rate is 1.44 × 10^5^°C/min at 50°C, which is a 12.5% increase, and the rate is just above the threshold (Figure S10C,D). A higher‐temperature rewarming solution can increase the rewarming rate by increasing the temperature gradient in Equation (1), thereby improving the heat flux rate. However, a few species of coral larvae with large volumes cannot reach the rewarming rate threshold even with a higher‐temperature rewarming solution or shearing embryos due to their large thermal mass (Figure S11), requiring other rewarming methods, such as laser rewarming [23, 29], to further increase the rewarming rate. Overall, the heat transfer model results demonstrate that optimizing larval volume and external rewarming conditions are essential for scalable cryopreservation of coral larvae with different volumes.

### 3.5 Banking

Two species, *L. scutaria* and *F. fungites*, had good survival at 24 h post‐thaw and were banked (see Table 3 for details). In 2022, the first coral larvae were banked at the Australian Institute of Marine Science in Townsville, Queensland, Australia. *Fungia fungites* larvae were pulled from rearing tanks at D5; approximately 200 larvae were loaded onto each cryomesh and vitrified. The vitrified cryomeshes were put into 3D‐printed storage boxes that held 10 cryomeshes. Nine boxes were stored in dry shippers, and approximately 18 000 cryopreserved larvae were transported back to the Taronga CryoDiversity Bank in Sydney, New South Wales, Australia.

**TABLE 3:** Successful cryopreservation steps and banking for species tested. Summary of species examined in this study, if they vitrified on the cryomesh, if they were motile at 2 and 24 h post‐thaw, how many larvae were banked, and where that cryobank is located.

Species	Vitrification on Mesh (Y/N)	Post‐thaw Motility 2 h (Y/N)	Post‐thaw Motility 24 h (Y/N)	Banked Larvae and Storage Location
***Lobactis scutaria***	Y	Y	Y	2024: 8900 larvae 2025: 63 400 larvae SNZCBI; USDA
***Fungia fungites***	Y	Y	Y	2022: 18 000 larvae Taronga
***Pocillopora meandrina***	Y	Y	N	N/A
***Dendrogyra cylindrus***	Y*	N*	N*	2024: 2100 larvae (not sheared) TFA

*Sheared Larvae

USDA = National Animal Germplasm Program at the United States Department of Agriculture

SNZCBI = Smithsonian National Zoo and Conservation Biology Institute

Taronga = Taronga Conservation Society Australia

TFA = the Florida Aquarium.

In 2024, *L. scutaria* larvae were cryopreserved at the Hawaiʻi Institute of Marine Biology. Eight unique genotypes were individually banked with an initial goal of 16 cryomeshes with 50 larvae per mesh per genotype. The number of cryomeshes per genotype varied depending on the quantity of larvae available; however, ∼8900 D4 and D5 larvae were banked (Table 3). These 2024 cryopreserved larvae were shipped to the USDA National Animal Germplasm Program biorepository in Fort Collins, Colorado, USA. Additionally, in 2024, approximately 8000 whole (unsheared) *D. cylindrus* larvae were cryopreserved and remain at The Florida Aquarium (Table 3).

In 2025, *L. scutaria* larvae were again banked using the cryomesh, but this time genotypes from each month were pooled (7 genotypes in July 2025 and 6 genotypes in August 2025). Approximately 200 larvae were loaded onto each cryomesh. Pooled *L. scutaria* larvae were cryopreserved on D3, D4, D5, and D6. Approximately 63 000 cryopreserved larvae from 317 cryomeshes in 2025 were sent to the Smithsonian Institution's coral biorepository in Front Royal, Virginia, USA (Table 3).

## 4 Discussion

In the marine ecosystem, cryopreservation is a nascent conservation tool that can help augment endangered coral populations. The first proof‐of‐concept studies demonstrated that cryopreserved coral sperm could produce coral larvae that successfully settled, signifying competency in a laboratory‐reared coral [45]. Subsequently, cryopreserved sperm from the brain coral *Diploria labyrinthiformis* were used to create coral larvae, and the developing larvae were settled and outplanted onto the Puerto Morelos Reef National Park [46], establishing that cryopreservation can be an active conservation tool. The work described here is another example of using cryopreservation to help reef populations by securing the genetic diversity of the endangered *D. cylindrus*, thereby reducing the extinction threat for this coral. Although we banked whole larvae of *D. cylindrus*, as methods advanced, we could thaw them differently to help ensure survival. Here we demonstrated that cryopreserved coral larvae showed normal morphology (though reduced in size) and swimming behavior post‐thaw, were competent to settle, and we also put larvae into a biorepository. Future experiments should include tracking post‐thaw settlement and growth of coral recruits in both an aquarium setting, as was done for the control (untreated) sheared larvae (Table 2 and Figure 5), and on the reef to monitor growth and maturation.

A global network of coral biorepositories could serve as an insurance policy for the ocean [28]. Because biodiversity is distributed among nations, such a system must respect national sovereignty while encouraging international cooperation. Regional facilities across the world's oceans could collect and store genetic material from local coral species, with redundant backups to ensure long‐term security. Table 3 shows the wide variety of locations where coral larvae were banked during these studies and supports the idea of having regional or nodal cryorepositories. With expanded funding, research, and coordination, conservationists could secure much of the remaining genetic diversity of reef‐building corals within years. There are more than 1000 species of Scleractinian coral; we cannot save them all. Difficult decisions about priorities will be necessary, but preserving as much genetic diversity as possible would make future restoration still feasible.

One of the main challenges for coral cryopreservation is having sufficient, high‐quality larvae that can withstand the stresses of the whole process. Reefs that have experienced multiple warming and subsequent bleaching events (and pressures from localized stressors) may not have reproductive material that is sufficiently robust to withstand the larval vitrification process. Larval quality and quantity may also be influenced by insufficient genetic diversity of parental colonies from which to collect gametes, desynchronized spawning that reduces the genotypes represented in the pool, poor fertilization success due to stress and disease, and population extirpation due to multiple stressors. While starting with robust, healthy larvae is undoubtedly important, other factors that support successful cryopreservation of coral larvae are larval volume and developmental stage, the concentration and type of cryoprotectant used, membrane permeability to water and cryoprotectants, toxicity to the cryoprotectants, the amount of fat in the larvae, symbiont status and menthol/light bleaching stress, husbandry differences, and the warming temperatures used.

A central result of the work presented here was that the small volume of the larvae alone did not predict success. For example, *P. meandrina* had small larvae and vitrified but did not survive to 24 h, and sheared *D. cylindrus* achieved smaller volumes that did not yield 24 h post‐thaw movement either. Across these studies, we assumed that the vitrification solution, PDT, permeated coral cells and tissues uniformly across all species. We made this assumption based on two factors: 1) PDT had already demonstrated early success in two fungiid species resulting in 24 h+ swimming, and 2) a relatively uniform freezing protocol using 10% DMSO successfully cryopreserves most species of coral sperm. However, this was likely not a reasonable assumption. When we looked more carefully, even though PDT produced swimming larvae in those two fungiid species, many of those larvae were fractured, suggesting that sub‐lethal ice formed, cracking but not killing them. This could have been caused by insufficient amounts of PDT entering the larvae, an inadequate warming rate, and/or devitrification in the rehydration medium; using a full‐strength vitrification solution instead of 0.5 m trehalose in FSW for the rapid warming step might offset the potential for devitrification.

We did test different exposure times to PDT to account for different larval volumes, but nevertheless, few of the larger‐volume species tested swam at 24 h. This may indicate that the equilibration times used were insufficient to achieve effective dehydration and cryoprotectant permeability in these larger larvae, but may also suggest that there are species differences in permeability that reduce the effectiveness of PDT in achieving vitrification. Moreover, larvae with symbionts had to be bleached, which added an additional stress, and husbandry differences or suboptimal larval rearing conditions may induce stress and reduce physiological fitness. This is particularly true for *P. meandrina*, which was difficult to maintain in larval culture. Finally, developmental age may also present a barrier. *Lobactis scutaria* were permeable to DMSO and PG on developmental D2‐D4, but by D5 they showed reduced permeability to those cryoprotectants [23]. Essential next steps should include toxicity measurements encompassing a suite of cryoprotectants in varying concentrations and exposure times, accompanied by shrink‐swell experiments to determine permeability rate; if successful vitrification and warming are achieved, those vitrified larvae need to settle and growth should be tracked.

This work is meant to serve as a helpful guide for the cryopreservation of other larval coral species. The first step in this process is to measure egg volume, which provides a sufficient approximation of larval volume and can be used to determine whether embryo shearing is required to bring that volume within the range required for cryomesh warming. For new species, this should be followed by exposure of larvae to a panel of cryoprotectants at a range of concentrations (e.g., 0.5–7 m) to determine which of those may be suitable for use in a vitrification solution – more than likely, a suite of non‐toxic cryoprotectants forming a vitrification cocktail will be needed. Cryoprotectants showing minimal toxicity can then be tested in shrink‐swell experiments to determine the exposure times required to achieve cellular dehydration and penetration. Combinations of cryoprotectants with minimal toxicity can then be tested at a range of dehydration and rehydration times to assess vitrification solution toxicity, and finally, vitrification experiments can proceed with the refined vitrification solutions and exposure timings. Both toxicity and vitrification experiments should include post‐treatment assessment of larval morphology and swimming behavior, and if possible, the surviving larvae can also be settled and grown out to monitor the effects of cryoprotectant exposure on development during early life stages (Figure 13). As evidenced by the present study, this approach is not a guarantee of success but will generate a thorough understanding of the cryobiology of the target species and provide a basis for continued experimentation and protocol refinement.

**FIGURE 13:**
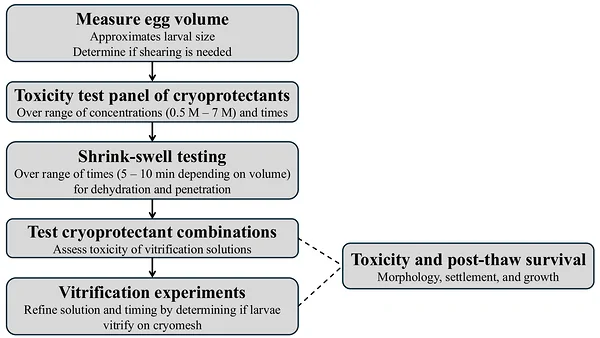
Flowchart of steps for coral larvae cryomesh vitrification. Knowing the egg/larval volume will help determine if a species is too large for cryomesh vitrification and if reducing the size (via shearing) is needed. Testing individual cryoprotectant (CPA) toxicity and shrink‐swell timing will guide which CPAs and concentrations should be explored for a vitrification solution. Testing the toxicity and vitrification success will refine the solution and protocol, and surviving larvae can then be assessed for morphology and monitored for any possible long‐term effects.

One of the challenging issues with these vitrification data was the fracturing of the coral larvae after vitrification that produced smaller, and possibly more, larvae than the untreated controls. While we did actively exclude small and irregular fragments, it is possible that the post‐thaw percent survival numbers may have been skewed by this, but it is likely consistent across all vitrification treatments. To better understand the effect of larval fracturing on survival numbers, we carefully counted and measured the volumes of hundreds of untreated controls and post‐thaw swimming larvae after vitrification. This yielded a robust distribution of the volumes produced by the cryopreservation processes and demonstrated that brief warming in a 50°C solution produced a median volume distribution closer to the controls than the 26°C warming (Figure 11). However, there was still a nearly 50% decrease in volume, and it did not address how to accurately assess post‐thaw survival numbers from vitrification. A future experimental goal to improve our overall vitrification process would aim for post‐thaw volumes that more closely match the control volume distribution.

In some species, successful vitrification was observed (i.e., no ice formed during freezing), and larvae showed movement at 2 h but then demonstrated no swimming at 24 h. The reasons for this are difficult to determine but might indicate that warming was not fast enough to prevent the formation of lethal ice crystals (i.e., devitrification). To investigate this, the embryos from some species with larger coral larvae were dissociated during early development to generate larvae with a reduced volume to improve heat transfer during warming. Cryopreservation of these smaller larvae did not result in swimming larvae at 24 h, indicating that other factors are involved in addition to the relationship between larval size and heat transfer. One possible important factor may be the amount of lipids in the larval tissues, since lipids play a role in the formation of intracellular ice [47]. Supporting this view were a handful of much larger *O. faveolata* larvae that were cryopreserved at 30+ days of age (i.e., when lipid reserves were depleted) and had fractured pieces swimming after 24 h post‐thaw. Further investigations are required to elucidate the roles of lipid content and larval age in the recovery of larvae from cryopreservation using cryomesh technology.

Importantly, reducing the volume of the coral larvae was not an impediment to their growth or settlement. Untreated *D. cylindrus* larvae that were sheared at the 2‐ to 8‐cell stage were able to settle, develop, and grow into normal mature coral (Figure 5). This suggests that the production of smaller larvae or pieces of swimming larvae, either by shearing or as a result of vitrification and warming, will not be an issue for future normal coral development and growth. Despite this, a better understanding of the impacts of shearing on the genetic distribution of the resulting larval culture, and whether there is more potential for chimerism due to the increased proportion of larvae with a high level of relatedness [48], would need to be determined before this approach could be implemented in a conservation setting. Finally, because some of these data were collected during a training workshop and our methods were not fully developed, there was some variability in the approaches taken to collect the data. The use of embryo dissociation to generate larvae for cryopreservation requires further work to standardize and improve the efficiency of the current methods but, nevertheless, may be key to the future success of larval cryopreservation and biobanking.

Currently, successful cryopreservation for a particular larval volume resides below the hatched line in Figure 1. Differences in larval volume were observed across development days in some species, some of which was likely due to sample size while for others it may have been due to standing variation. However, in general, the egg volume provided a reasonable proxy for the expected size of the larvae. Therefore, when assessing future species for cryopreservation, the egg can be assessed quickly and easily as soon as the coral spawns, and further steps can be taken to shear the larvae early in development if needed. In some of the sheared larval batches, an increase in the measured larval size was observed at D3 and D4. The specific reason for this is unknown but may have been related to developmental changes such as gastrulation, fusion of sheared larvae during rearing that produced larger volumes, or sampling effects due to preferential selection of smaller larvae at earlier stages. Variability in the size of sheared *D. cylindrus* larvae, as well as the apparently higher lipid content of this species compared to others tested, may have been factors in the failure of cryopreservation in this treatment. Thus, care must be taken to ensure the use of uniformly sized sheared larvae on a particular date and, if possible, waiting until later developmental dates when lipid reserves may be depleted, potentially leading to increased cryopreservation success (as in *O. faveolata*).

The heat transfer modeling results highlight the critical role of larval size and geometry in cryomesh vitrification‐based cryopreservation. Larger larvae exhibited lower theoretical rewarming rates due to increased thermal resistance, increasing the risk of devitrification during rewarming. In contrast, mechanically sheared larvae achieved substantially higher rewarming rates because of their reduced effective volume and smaller thermal mass. The model also demonstrated that increasing the external rewarming temperature significantly improved rewarming rate across all species and developmental stages. Overall, these findings emphasize that optimizing larval size and heat transfer conditions are essential for scalable cryopreservation of coral larvae, potentially enabling reduced CPA concentrations and lower toxicity.

It is essential to focus on specific critical parameters to improve the achievable cooling rate, such as using materials with higher thermal conductivity for the cryomesh. Additionally, thermal mass can be reduced by using thinner wire diameters and minimizing the nitrogen vapor barrier through vertical plunging to allow the release of the vapor barrier from the cryomesh. Increased cooling rates enable the use of lower cryoprotectant concentrations, lowering the associated toxicity, which can help facilitate the cryopreservation of biosystems that have not yet been preserved due to susceptibility to cryoprotectants, such as coral larvae [24]. With the improved cooling rate and vertical plunge, uniform cooling was achieved for larger cryomeshes (e.g., 15 × 4 cm) by using thermally conductive materials and optimized cryomesh design [24]. The scale‐up achieved by conduction‐dominated cryomesh paves the way to cryopreserve a wide range of organisms in greater quantities.

## 5 Conclusions

A roadmap to cryopreserve coral larvae was presented using conduction‐mediated cryomesh technology that is easy to make and use under field conditions. The volumes of eggs and embryos from many species of coral were measured as one factor that may help determine the efficacy of heat transfer on the cryomesh. Only coral larvae from two fungiid species that were aposymbiotic and had a volume of ∼0.002 mm^3^ demonstrated post‐thaw swimming at 24 h. Small volume larvae with symbionts (*P. meandrina*) were cryopreserved with little success, possibly because of the stress of the bleaching process to remove the symbionts, the husbandry techniques to rear the larvae, poor cryoprotectant permeability or cryoprotectant toxicity. Heat transfer modeling further demonstrated that reduced larval volume significantly increased achievable rewarming rates by shortening conductive heat transfer distances, supporting the improved cryopreservation performance observed in smaller or mechanically sheared larvae. Cryopreservation of *D. cylindrus* larvae sheared at the 2‐ to 8‐cell stage to produce smaller larval fragments ∼0.002 mm^3^ did not produce post‐thaw swimming larvae for potentially many reasons; however, these smaller untreated fragments were competent to settle and mature into normal coral. Therefore, as this cryopreservation process advances, reducing larval size by embryonic dissociation to improve heat transfer would not be an impediment to producing competent larvae with the potential to use these smaller post‐thaw larvae in restoration efforts.

Next steps to improve this cryopreservation process should include examining the permeability and toxicity of different cryoprotectants, conducting shrink‐swell experiments to understand water and cryoprotectant permeability and toxicity across many species and larval sizes, improving husbandry to allow the larvae or fragments to use up much of their fats without impacting their overall health and survival, and using a wide variety of improved heat transfer techniques and increased warming to improve overall vitrification and ultimately conservation success.

## Author Contributions

**Mary Hagedorn** (conceptualization; visualization; writing – original draft; writing – review & editing), **E. Michael** **Henley** (experimental planning, data collection and coordination, statistical analysis and results, writing – review, and editing), **Jonathan Daly** (experimental planning, data collection, writing – review and editing). **Kristen Marhaver** and **Chris Lippens** helped plan and conduct research in Florida and Curaçao, contributed data, and reviewed the manuscript. **Anastazia Banaszak** and **Victoria Grosso** helped plan and conduct research in Florida and Mexico, contributed data, and reviewed the manuscript. **Frania Valdez** helped conduct research in Mexico, **Emily Williams** (coral husbandry, data collection, writing – review, and editing). **Keri O'Neil** helped plan and conduct workshops in Florida, oversaw coral husbandry and spawning activities, and reviewed the manuscript. **Nikolas Zuchowicz** assisted in data collection, ImageJ macro development, and review of the manuscript. **Zongqi Guo** (visualization, data collection, modeling, writing – review, and editing).

## Funding

G20 Coral Research & Development Accelerator Platform – CAP‐2022‐1193; The Paul M. Angell Family Foundation – CON‐F24‐31; Revive & Restore – 2023–049; The Volgenau Foundation; The Zegar Family Foundation; The Anela Kolohe Foundation; The Cedar Hill Foundation; Pew Fellowship in Marine Conservation; Carlos Slim Foundation and SECORE International; National Science Foundation Engineering Research Center: Advanced Technologies for the Preservation of Biological Systems (Grant No. EEC 1941543); USF Foundation and the USF Research Foundation; Reef Restoration and Adaptation Program, The Taronga Conservation Science Initiative, and The Taronga Foundation.

## Conflicts of Interest

Z.G. and N.Z. are inventors on a pending patent application (WO2024123869). Z.G. is an equity holder in a company that is commercializing the cryomesh technology discussed in this article. All other authors declare no conflicts.

## Supporting information




**Supporting File 1**: advs78550‐sup‐0002‐SuppMat.docx.

**Supporting File 2**: advs78550‐sup‐0001‐SuppMat.xlsx.

## Data Availability

The data that supports the findings of this study are available in the supplementary material of this article.
