# Supplementary material for: Steps Toward Vitrification and Banking of Coral Larvae Using Field‐Friendly Cryomesh Technology
Source: Adv Sci (Weinh). 2026 Nov 10:e78550. Online ahead of print. doi: 10.1002/advs.78550 (PMC13749476; doi:10.1002/advs.78550)
Supplement: Supplementary file 1 [file ADVS-9999-e78550-s002.docx]

**Supplemental Information for**

**Steps Toward Vitrification and Banking of Coral Larvae Using Field-Friendly Cryomesh Technology**

*E. Michael Henley, Jonathan Daly, Claire Lager, Nikolas Zuchowicz, Riley Perry, Jaylyn Oka, Mariko Quinn, Zongqi Guo, Maria Victoria Grosso Becerra,* *Frania Valdez Felix,* *Anastazia T. Banaszak, Christian R.A. Lippens, Kristen L. Marhaver, Emily Jane Williams, Keri O’Neil, and Mary Hagedorn*

Send correspondence to [HenleyM@si.edu](mailto:HenleyM@si.edu)

**This file includes:**

Tables S1 to S5:

- Additional coral egg and larval volumes
- Descriptive statistics for larval toxicity percent survival
- Descriptive statistics for larval cryopreservation percent survival
- Descriptive statistics for the volumes of L. scutaria larvae warmed at 26 °C and 50 °C
- Physical properties for heat transfer simulation

Figures S1 to S7

- Images of 3D-printed “cryobox” and cryomesh
- Volumes and developmental day of eggs and unsheared and sheared larvae for additional coral species

Detailed description of the vitrification and rewarming model developed for this study

Figures S8 to S11

- Theoretical cooling and rewarming rates of coral larvae under varying volumes, developmental days, and temperatures from that model

Instructions for ImageJ macro and measurements for eggs and larvae

**Additional supplementary information for this manuscript:**

“Supplemental_Data” is an Excel file that includes the raw data for this study. Each tab corresponds to:

1. Raw data for egg and larval volumes
2. Raw data for toxicity percent survival
3. Raw data for cryopreservation post-thaw percent survival
4. Raw data for the control and post-thaw volumes of *L. scutaria* larvae warmed at 26 °C and 50 °C
5. Results of statistical tests

**Table S1. Volume (mm^3^) of coral eggs and larvae across developmental days, continued.**

Eggs or larvae were imaged on the day of spawning (D0, eggs) through different developmental days. The sheared larvae were broken apart at the 2-8 cell stage the night of spawning, and their volume measurements began on D1. Measurements were recorded on a single day for each of these larvae: *P. acuta* (brooding species), *O. faveolata* (Curaçao, 36 days old), *A. spathulata* (D3). The sample size (n) refers to the number of eggs or larvae measured at each timepoint.

| ***M. angulosa*** | **Day 0** | **Day 1** | **Day 2** | **Day 3** | **Day 4** | **Day 5** |
| --- | --- | --- | --- | --- | --- | --- |
| Genotypes | 7 | 7 | 7 | 7 | n/a | n/a |
| n | 10 | 10 | 10 | 10 | n/a | n/a |
| Median (mm^3^) | 0.1761 | 0.1792 | 0.1415 | 0.1753 | n/a | n/a |
| Mean (mm^3^) | 0.1948 | 0.1855 | 0.1504 | 0.1779 | n/a | n/a |
| SD | 0.0441 | 0.0562 | 0.0242 | 0.0369 | n/a | n/a |
| SEM | 0.0139 | 0.0178 | 0.0077 | 0.0117 | n/a | n/a |
|  |  |  |  |  |  |  |
| ***M. angulosa - Sheared*** | **Day 0** | **Day 1** | **Day 2** | **Day 3** | **Day 4** | **Day 5** |
| Genotypes | n/a | 7 | 7 | 7 | 7 | n/a |
| n | n/a | 10 | 10 | 10 | 10 | n/a |
| Median (mm^3^) | n/a | 0.0168 | 0.0166 | 0.0597 | 0.0470 | n/a |
| Mean (mm^3^) | n/a | 0.0173 | 0.0176 | 0.0514 | 0.0497 | n/a |
| SD | n/a | 0.0053 | 0.0063 | 0.0207 | 0.0180 | n/a |
| SEM | n/a | 0.0017 | 0.0020 | 0.0066 | 0.0057 | n/a |
|  |  |  |  |  |  |  |
| ***C. natans*** | **Day 0** | **Day 1** | **Day 2** | **Day 3** | **Day 4** | **Day 5** |
| Genotypes | 2 | 2 | 2 | n/a | n/a | n/a |
| n | 10 | 10 | 10 | n/a | n/a | n/a |
| Median (mm^3^) | 0.0291 | 0.0292 | 0.0321 | n/a | n/a | n/a |
| Mean (mm^3^) | 0.0288 | 0.0292 | 0.0325 | n/a | n/a | n/a |
| SD | 0.0020 | 0.0034 | 0.0036 | n/a | n/a | n/a |
| SEM | 0.0006 | 0.0011 | 0.0011 | n/a | n/a | n/a |
|  |  |  |  |  |  |  |
| ***C. natans - Sheared*** | **Day 0** | **Day 1** | **Day 2** | **Day 3** | **Day 4** | **Day 5** |
| Genotypes | n/a | 2 | 2 | 2 | 2 | n/a |
| n | n/a | 10 | 10 | 10 | 10 | n/a |
| Median (mm^3^) | n/a | 0.0030 | 0.0045 | 0.0029 | 0.0034 | n/a |
| Mean (mm^3^) | n/a | 0.0028 | 0.0044 | 0.0035 | 0.0034 | n/a |
| SD | n/a | 0.0006 | 0.0018 | 0.0018 | 0.0010 | n/a |
| SEM | n/a | 0.0002 | 0.0006 | 0.0006 | 0.0003 | n/a |
|  |  |  |  |  |  |  |
| ***P. strigosa*** | **Day 0** | **Day 1** | **Day 2** | **Day 3** | **Day 4** | **Day 5** |
| Genotypes | 4 | 4 | 4 | 4 | n/a | n/a |
| n | 10 | 10 | 10 | 10 | n/a | n/a |
| Median (mm^3^) | 0.0343 | 0.0350 | 0.0448 | 0.0420 | n/a | n/a |
| Mean (mm^3^) | 0.0356 | 0.0345 | 0.0451 | 0.0443 | n/a | n/a |
| SD | 0.0045 | 0.0047 | 0.0077 | 0.0100 | n/a | n/a |
| SEM | 0.0014 | 0.0015 | 0.0024 | 0.0032 | n/a | n/a |
|  |  |  |  |  |  |  |
| ***P. strigosa - Sheared*** | **Day 0** | **Day 1** | **Day 2** | **Day 3** | **Day 4** | **Day 5** |
| Genotypes | n/a | 4 | 4 | 4 | 4 | n/a |
| n | n/a | 10 | 10 | 10 | 10 | n/a |
| Median (mm^3^) | n/a | 0.0035 | 0.0043 | 0.0056 | 0.0057 | n/a |
| Mean (mm^3^) | n/a | 0.0037 | 0.0056 | 0.0059 | 0.0057 | n/a |
| SD | n/a | 0.0011 | 0.0029 | 0.0029 | 0.0019 | n/a |
| SEM | n/a | 0.0004 | 0.0009 | 0.0009 | 0.0006 | n/a |
|  |  |  |  |  |  |  |
| ***D. labyrinthiformis*** | **Day 0** | **Day 1** | **Day 2** | **Day 3** | **Day 4** | **Day 5** |
| Genotypes | 3 | n/a | n/a | 3 | 3 | n/a |
| n | 11 | n/a | n/a | 10 | 11 | n/a |
| Median (mm3) | 0.0444 | n/a | n/a | 0.0337 | 0.0322 | n/a |
| Mean (mm3) | 0.0451 | n/a | n/a | 0.0334 | 0.0332 | n/a |
| SD | 0.0036 | n/a | n/a | 0.0030 | 0.0064 | n/a |
| SEM | 0.0011 | n/a | n/a | 0.0009 | 0.0019 | n/a |
|  |  |  |  |  |  |  |
| ***O. faveolata*** | **Day 0** | **Day 1** | **Day 2** | **Day 3** | **Day 4** | **Day 5** |
| Genotypes | 2 | n/a | 2 | 2 | n/a | n/a |
| n | 12 | n/a | 11 | 11 | n/a | n/a |
| Median (mm3) | 0.0177 | n/a | 0.0165 | 0.0172 | n/a | n/a |
| Mean (mm3) | 0.0187 | n/a | 0.0164 | 0.0166 | n/a | n/a |
| SD | 0.0031 | n/a | 0.0025 | 0.0235 | n/a | n/a |
| SEM | 0.0009 | n/a | 0.0008 | 0.0007 | n/a | n/a |
|  |  |  |  |  |  |  |
| ***O. annularis*** | **Day 0** | **Day 8** | **Day 9** |  |  |  |
| Genotypes | 4 | 4 | 4 |  |  |  |
| n | 11 | 10 | 12 |  |  |  |
| Median (mm3) | 0.0244 | 0.0094 | 0.0171 |  |  |  |
| Mean (mm3) | 0.0245 | 0.0115 | 0.0181 |  |  |  |
| SD | 0.0027 | 0.0105 | 0.0046 |  |  |  |
| SEM | 0.0008 | 0.0033 | 0.0013 |  |  |  |
|  |  |  |  |  |  |  |
| **Larval Volume - Single Day Sampling** | | | | |  |  |
| **Species** | ***P. acuta*** | ***P. acuta* - Cut** | ***A. spathulata*** | ***O. faveolata*** |  |  |
| Genotypes | 6 | 5 | 7 | n/a |  |  |
| n | 60 | 50 | 9 | 18 |  |  |
| Median (mm^3^) | 0.3425 | 0.0287 | 0.1028 | 0.0195 |  |  |
| Mean (mm^3^) | 0.3649 | 0.0349 | 0.1014 | 0.0215 |  |  |
| SD | 0.1879 | 0.0249 | 0.0121 | 0.0076 |  |  |
| SEM | 0.0243 | 0.0035 | 0.0040 | 0.0018 |  |  |

**Table S2. Descriptive statistics for toxicity exposure percent survival.**

Larvae from these species were exposed to PDT to examine their exposure limits. *Lobactis scutaria* and *D. cylindrus* were tested on different developmental days and three exposure times while *P. meandrina* and *F. fungites* were tested on D4. *Pocillopora meandrina* was tested at two exposure times, and rehydration in 0.5 M trehalose was the same as exposure time for *L. scutaria*, *D. cylindrus*, and *P. meandrina.* *Fungia fungites* was examined with different dehydration / rehydration scenarios. For all species and treatments, n=number of trials.

| **Toxicity: Percent Survival ~ Development Day x Exposure Time** | | | | | | | | | | | | |
| --- | --- | --- | --- | --- | --- | --- | --- | --- | --- | --- | --- | --- |
| ***L. scutaria*** | **60 s** | | | | **90 s** | | | | **120 s** | | | |
| Develop Day | Genotypes | Mean (%) | SD | n | Genotypes | Mean (%) | SD | n | Genotypes | Mean (%) | SD | n |
| Day 2 | 9 | 0.7467 | 0.0706 | 9 | 9 | 0.791 | 0.0988 | 9 | 9 | 0.871 | 0.0482 | 9 |
| Day 3 | 14 | 0.7851 | 0.1566 | 14 | 6 | 0.845 | 0.1282 | 6 | 6 | 0.816 | 0.1643 | 6 |
| Day 4 | 7 | 0.7107 | 0.1191 | 7 | 7 | 0.683 | 0.1680 | 7 | 7 | 0.702 | 0.1361 | 7 |
| Day 5 | 12 | 0.7771 | 0.1386 | 12 | 6 | 0.818 | 0.0516 | 6 | 3 | 0.752 | 0.2595 | 3 |
|  |  |  |  |  |  |  |  |  |  |  |  |  |
| **Toxicity: Percent Survival ~ Development Day** | | | | |  |  |  |  |  |  |  |  |
| ***L. scutaria*** | Genotypes | Mean (%) | SD | n |  |  |  |  |  |  |  |  |
| Day 2 | 27 | 0.783 | 0.089 | 27 |  |  |  |  |  |  |  |  |
| Day 3 | 26 | 0.818 | 0.149 | 26 |  |  |  |  |  |  |  |  |
| Day 4 | 21 | 0.692 | 0.136 | 21 |  |  |  |  |  |  |  |  |
| Day 5 | 21 | 0.785 | 0.136 | 21 |  |  |  |  |  |  |  |  |
|  |  |  |  |  |  |  |  |  |  |  |  |  |
| **Toxicity: Percent Survival ~ Development Day x Exposure Time** | | | | | | | | | | | | |
| ***D. cylindrus*** | **60 s** | | | | **90 s** | | | | **120 s** | | | |
| Develop Day | Genotypes | Mean (%) | SD | n | Genotypes | Mean (%) | SD | n | Genotypes | Mean (%) | SD | n |
| Day 2 | 14 | 0.617 | 0.1533 | 4 | 20 | 0.543 | 0.3919 | 4 | 20 | 0.488 | 0.1906 | 4 |
| Day 3 | 22 | 0.469 | 0.1889 | 7 | 20 | 0.433 | 0.2538 | 4 | 20 | 0.349 | 0.1348 | 4 |
|  |  |  |  |  |  |  |  |  |  |  |  |  |
| **Toxicity: Percent Survival ~ Exposure Time** | | | | |  |  |  |  |  |  |  |  |
| ***P. meandrina*** | Genotypes | Mean (%) | SD | n |  |  |  |  |  |  |  |  |
| 30 seconds | 6 | 0.7588 | 0.1962 | 9 |  |  |  |  |  |  |  |  |
| 60 seconds | 6 | 0.6737 | 0.2655 | 12 |  |  |  |  |  |  |  |  |
|  |  |  |  |  |  |  |  |  |  |  |  |  |
| **Toxicity: Percent Survival ~ Dehyd. / Rehyd. Time** | | | | |  |  |  |  |  |  |  |  |
| ***F. fungites*** | Genotypes | Mean (%) | SD | n |  |  |  |  |  |  |  |  |
| 60s / 60s | 7 | 0.34 | 0.0424 | 2 |  |  |  |  |  |  |  |  |
| 120s / 60s | 7 | 0.36 | 0.1414 | 2 |  |  |  |  |  |  |  |  |
| 60s / 120s | 7 | 0.54 | 0.0282 | 2 |  |  |  |  |  |  |  |  |

**Table S3. Descriptive statistics for cryopreservation post-thaw percent survival.**

Larvae from *L. scutaria* and *F. fungites* were vitrified with PDT on cryomesh, and their survival was assessed at 24 h after warming. *Lobactis scutaria* vitrification was tested on developmental D4 and D5 at three exposure times, while F. *fungites* was tested with the same dehydration / rehydration scenarios as its toxicity trials. These were warmed at 26 °C in 0.5 M trehalose. Another subset of vitrified D5 *L. scutaria* larvae were briefly warmed at 50 °C in 0.5 M trehalose and then placed into 26 °C 0.5 M trehalose for the remainder of their rehydration time. For all species and treatments, n = number of trials.

| **Cryopreservation: Percent Survival ~ Development Day x Exposure Time** | | | | | | | | | | | | |
| --- | --- | --- | --- | --- | --- | --- | --- | --- | --- | --- | --- | --- |
| ***L. scutaria*** | **60 s** | | | | **90 s** | | | | **120 s** | | | |
| Develop Day | Genotypes | Mean (%) | SD | n | Genotypes | Mean (%) | SD | n | Genotypes | Mean (%) | SD | n |
| Day 4 | 14 | 0.581 | 0.362 | 7 | 7 | 0.465 | 0.314 | 7 | 12 | 0.503 | 0.270 | 12 |
| Day 5 | 6 | 0.717 | 0.078 | 6 | 6 | 0.772 | 0.095 | 6 | 6 | 0.488 | 0.117 | 6 |
|  |  |  |  |  |  |  |  |  |  |  |  |  |
| **Cryopreservation: Percent Survival ~ Warming Temp x Exposure Time** | | | | | | | | | | | | |
| ***L. scutaria* (D5)** | **60 s** | | | | **90 s** | | | | **120 s** | | | |
| Warming Temp | Genotypes | Mean (%) | SD | n | Genotypes | Mean (%) | SD | n | Genotypes | Mean (%) | SD | n |
| 25 °C | 6 | 0.717 | 0.078 | 6 | 6 | 0.772 | 0.095 | 6 | 6 | 0.488 | 0.117 | 6 |
| 50 °C | 4 | 0.821 | 0.053 | 5 | 5 | 0.815 | 0.122 | 5 | 6 | 0.665 | 0.135 | 6 |

| **Cryopreservation: Percent Survival ~ Dehyd. / Rehyd. Time** | | | | |
| --- | --- | --- | --- | --- |
| ***F. fungites*** | Genotypes | Mean (%) | SD | n |
| 60 s / 60 s | 7 | 0.50 | 0.311 | 2 |
| 120 s / 60 s | 7 | 0.72 | 0.099 | 2 |
| 60 s / 120 s | 7 | 0.30 | 0.110 | 2 |

**Table S4. Descriptive statistics for the volume of *L. scutaria* larvae warmed at two temperatures.**

In addition to post-thaw survival, the volumes of D5 *L. scutaria* larvae warmed at 26 °C and 50 °C (both 0.5 M trehalose) were compared to one another as well as the volume of D5 control larvae.

| **Larval Volume: Day 5 *L. scutaria* Warming Temp** | | | |
| --- | --- | --- | --- |
| **Treatment** | **Control** | **26 °C** | **50 °C** |
| N (genotypes) | 6 | 6 | 6 |
| n | 99 | 221 | 239 |
| Median (mm^3^) | 0.0021 | 0.0007 | 0.0010 |
| Mean (mm^3^) | 0.0023 | 0.0007 | 0.0011 |
| SD | 0.0008 | 0.0004 | 0.0006 |
| SEM | 0.00008 | 0.00003 | 0.00004 |

**Figure S1. 3D-printed insert (left) with capacity for 80 cryomesh frames (right).**

This insert was designed to fit inside the VWR CryoPro storage box, which is designed to fit liquid nitrogen freezer racks. The cryomesh consists of a 3D-printed frame that is attached to 35 μm stainless steel mesh. The specifications and schematics for these can be found in Lager et al.^[39]^

**Figure S2. Volume comparison of *M. angulosa* across early development, control and sheared larvae.**

**A)** *Mussa angulosa* larval volume was relatively consistent from initial spawning across three days of development, with no significant difference across days (p=0.1204). **B)** The sheared *M. angulosa* mean volume, however, did significantly change (p<0.0001), with the later days becoming larger, possibly from fusion of the sheared larvae. The decrease in the final volume from normal to sheared larval volume was approximately 72%. For both control and sheared larvae, these data were collected from one pool consisting of seven genotypes, and at each developmental day 10 larvae were imaged from this pool. Different letters above plots are statistically significant.

**Figure S3. Volume comparison of *C. natans* across early development, control and sheared larvae.**

**A)** *Colpophyllia natans* mean larval volume steadily and significantly increased from initial spawning across four days of development (p=0.0252). **B)** Sheared *C. natans* median volume changes during development, while significant (p=0.0491, just below the α = 0.05 threshold ), were relatively similar when comparing the initial and final volumes. The percentage change in volume from control to sheard decreased nearly 89%. For both control and sheared larvae, the data were collected from one pool consisting of two genotypes, and at each developmental day 10 eggs or larvae were imaged from this pool. Different letters above plots are statistically significant.

**Figure S4. Volume comparison of *P. strigosa* across early development, control and sheared larvae.**

**A)** Mean larval volume for *P. strigosa* was significantly larger during the last two days of development compared to the first two (p=0.0016). **B)** Sheared *P. strigosa* mean larval volume did not significantly differ across four developmental days (p=0.1266). There was an approximately 86% decline from control to sheared larval volume for this species. For both control and sheared larvae, the data were collected from one pool consisting of four genotypes, and at each developmental day 10 eggs or larvae were imaged from this pool. Different letters above plots are statistically significant.

**Figure S5. Volume comparison of *Diploria labyrinthiformis* across early development.**

**A)** Volume of eggs and developing *D. labyrinthiformis* larvae. **B)** Image of a Day 4 *D. labyrinthiformis* larva and **C)** and egg. Volume significantly declined from spawning throughout development (p<0.001). These data were collected from one pool consisting of three genotypes. At each developmental day, 10 eggs or larvae were imaged from the pool. The dotted line is the median, and different letters above plots indicate statistical significance.

**Figure S6. Volume comparison of *Orbicella annularis* across early development.**

**A)** Volume of eggs and developing *O. annularis* larvae. **B)** Image of a Day 9 *O. annularis* larva **C)** and egg. The volume decreased significantly across developmental days from spawning to Day 9 (p<0.001). These data were collected from one pool consisting of four genotypes. At each developmental day, ten eggs or larvae were imaged from the pool. The dotted line is the median, and different letters above plots indicate statistical significance.

**a**

**a**

**a**

**Figure S7. Volume comparison of *Orbicella faveolata* across early development.**

**A)** Volume of eggs and developing *O. faveolata* larvae. **B)** Image of a Day 3 *O. faveolata* larva and **C)** and egg. There was no significant change in volume from spawning across these developmental days (p=0.0905). These data were collected from one pool consisting of two genotypes, and at each developmental day, ten eggs or larvae were imaged from the pool. The dotted line is the median and shared letters above plots indicate no statistical significance.

**Modeling Vitrification and Rewarming Success Based on Volume**

This model describes the heat flux (*q"*) during cooling, which will have a linear relation with the heat loss rate and thus the cooling or rewarming rate. We describe these conditions through a simple 1D thermal resistance model (Figure 12A), which is

|  | $q^{''}= \frac{\Delta T}{R_{h}+R_{m}+R_{b}}= \frac{T_{i}-T_{\infty}}{R_{h}+R_{m}+R_{b}}=\frac{T_{i}-T_{\infty}}{R_{h,c}+R_{b,c}}$ | S1 |
| --- | --- | --- |

where *ΔT* is the temperature difference between the initial temperature of biosystem and final temperature (LN_2_ or rewarming solution), *R*_h_ is the external resistance of convection between the mesh and LN_2_, *R*_h,c_ is the external resistance of convection between the biosystem and LN_2_, *R*_m_ is the internal conduction thermal resistance of the mesh, and *R*_b_ and *R*_b,c_ are the conduction thermal resistance of the biosystem. Then, we simplified those three thermal resistances as:

|  | $R_{h}=\frac{1}{hA_{m}}$ | S2 |
| --- | --- | --- |
|  | $R_{m}=\frac{D}{k_{m}A_{cm}}$ | S3 |
|  | $R_{b}=\frac{L_{s}}{k_{b}A_{b}}$ | S4 |
|  | $R_{b,c}=\frac{L_{s,c}}{k_{b}A_{b}}$ | S5 |
|  | $R_{h,c}=\frac{L_{s,c}}{h_{c}A_{b}}$ | S6 |

where *h* and *h*_c_ are the convection heat transfer coefficient between the LN_2_ and cryomesh, *A*_m_ is the contact area between LN_2_ and cryomesh, *A*_cm_ is the cross-section area of the mesh, *D* is the mesh wire diameter, *k*_m_ is the thermal conductivity of the mesh, *k*_b_ is the thermal conductivity of the biosystem, *L*_s_ is the thickness of the biosystem, and *A*_b_ is the cross-section area of the biosystem. Based on the equations (Eqs. S1–6), a small thermal resistance leads to a high heat flux, which in turn corresponds to a higher cooling and rewarming rate.

During cryomesh cooling in LN_2_ or rewarming in rewarming solution, there are two processes of heat release: 1) convection heat transfer, which transfers the heat of the mesh and biosystem to liquid nitrogen (vitrification) or to the rewarming solution (rewarming), and 2) conduction heat transfer inside and between the mesh and biosystem. We further studied the heat transfer of conduction-dominated cryomesh in liquid nitrogen or from rewarming solution with a 1D transient heat transfer model (Figure 12A). Thus, the traditional governing heat transfer equation is

|  | $\frac{\partial T}{\partial t}= \alpha\frac{\partial^{2}T}{{\partial x}^{2}}$ | S7 |
| --- | --- | --- |

with boundary conditions

|  | $\frac{\partial T(0,t)}{\partial t}= 0$ | S8 |
| --- | --- | --- |
|  | $-k\frac{\partial T\left( L_{s},t \right)}{\partial x}=h[T\left( L_{s},t \right)-T_{\infty}]$ | S9 |

and the initial conditions of cooling

|  | ${T\left( x,0 \right)= T}_{i}=24-26 ^{\circ}C, T_{\infty}=-196 ^{\circ}C$ | S10 |
| --- | --- | --- |

and rewarming

|  | ${T\left( x,0 \right)= T}_{i}=-196 ^{\circ}C, T_{\infty}=24-26 or 50 ^{\circ}C$ | S11 |
| --- | --- | --- |

where *T* is the temperature; *t* is the time; *α* is the thermal diffusivity; *k* is the thermal conductivity; *L*_s_ is the thickness of the biosystem affected by mesh; and *L*_s,c_ is the thickness of the biosystem affected by direct liquid nitrogen cooling or rewarming of solution. We assumed a uniform initial temperature and environment temperature. Based on the transient heat transfer Eq. S7, we further simplified the heat transfer model to calculate the heat release time.

The heat release time is defined here as the time for the whole system (mesh with biosystem) to transfer to the desired temperature (e.g., LN_2_ temperature or rewarming solution temperature). We divided the total heat release time (*t*_total_) into two parts:

|  | $t_{total}= t_{c}+t_{m}+t_{b}=t_{h,c}+t_{b,c}$ | S12 |
| --- | --- | --- |

where *t*_c_ is the heat release time from mesh to outside (LN_2_ or rewarming solution) through convection heat transfer, *t*_m_ is the heat release time for conduction through the mesh, *t*_b_ is the heat release time for conduction through the biosystem, *t*_h,c_ is the heat release time to outside (LN_2_ or rewarming solution) through convection heat transfer without mesh contribution, and *t*_b,c_ is the heat release time for conduction through biosystem affected by convection only. Symmetry of zero heat flux at x = 0 as ∂T/∂x = 0. We calculated the heat release times similar to calculating a time constant (*τ*), which is

|  | $\tau= \frac{\rho c_{p}V}{hA}$ | S13 |
| --- | --- | --- |

where *ρ* is the density, *c*_p_ is specific heat, and *V* is the body volume. We assumed that all heat from the system (mesh with biosystem) is released from the mesh to LN_2_ through convection heat transfer. We calculated *t*_c_ as

|  | $t_{c}= \frac{Q_{m}+Q_{b}}{hA_{m}}$ | S14 |
| --- | --- | --- |

where *h* is the convection heat transfer coefficient with a range from 250 to 5000 W/m^2^/K, depending on cooling or rewarming. The heat of the mesh *Q*_m_ is

|  | $Q_{m}= \rho_{m}C_{p,m}V_{m}\Delta T$ | S15 |
| --- | --- | --- |
|  | $V_{m}= 4\pi\left( \frac{D}{2} \right)^{2}L_{u}$ | S16 |

where *D* is the mesh wire diameter (~30 µm), and *L*_u_ is the unit length (repeat length of cryomesh geometry and larvae geometry), which is *L*_u_ = 2(*D*+*P*). *P* is the pore size of the cryomesh. The heat of the biosystem *Q*_b_ is

|  | $Q_{b}= \rho_{b}C_{p,b}V_{b}\Delta T$ | S17 |
| --- | --- | --- |
|  | $V_{b}= L_{s}\cdot{L_{u}}^{2}$ | S18 |

where *L*_s_ is the thickness of the biosystem. Here, we treat the larvae as cubes to simplify the model and ensure uniform thickness. *A*_m_ is the contact area between the mesh and LN_2_, calculated as

|  | $A_{m}=(4\pi DL_{u}-4{\pi D}^{2})/2$ | S19 |
| --- | --- | --- |

The contact area considers one side of the surface area of the entire mesh and excludes the intersection area between each wire. We calculated *t*_m_ as

|  | $t_{m}= \frac{Q_{m}\cdot D}{{k_{m}A}_{cm}}$ | S20 |
| --- | --- | --- |

where *k*_m_ is the thermal conductivity of the mesh and the cross-section area of mesh was calculated as

|  | $A_{cm}=\Phi\cdot{L_{u}}^{2}$ | S21 |
| --- | --- | --- |

where *Ф* is the solid fraction (0.5-0.6). The *t*_b_ was then calculated as

|  | $t_{b}= \frac{Q_{b}\cdot L_{s}}{{k_{b}A}_{b}}$ | S22 |
| --- | --- | --- |

where *k*_m_ is the thermal conductivity of the mesh and *A*_b_ is the cross-section area as *L*_u_^2^.

To further simplify the convection effect for the biosystem exposed directly to LN_2_ or rewarming solution (the side away from mesh), we assumed the *h*_c_ was the same as on the mesh side. Note the experimental *h*_c_ for the biosystem might be lower than the assumption due to the nitrogen vapor layer (“Leidenfrost effect”). Thus, we can assume the time for heat release time *t*_b,c_ of biosystem through convection is

|  | $t_{b,c}=\frac{Q_{b,c}\cdot L_{s,c}}{{k_{b}A}_{b}}+\frac{Q_{b,c}}{h_{c}A_{b}}$ | S23 |
| --- | --- | --- |

where *Q*_b,c_ is the total heat of biosystem released by convection and *L*_s,c_ is the length of biosystem affected by convection heat transfer. For each measured larval volume V, total thickness was approximated by the diameter of an equal-volume sphere, Lt = (6V/π)^(1/3). As one example, the thickness of coral larvae was around 131 μm, with a volume of around 0.0012 mm^3^, we assigned *L*_t_ as the biosystem thickness of 131 μm. Then we combined Eqs. S12 and S23 to solve the cooling rate, where *t*_total_ *= t*_b,c_. By assuming *t*_total_ *= t*_b,c_, we calculated the total biosystem thickness, which is

|  | $L_{t}={L_{s}+L}_{s,c}$ | S24 |
| --- | --- | --- |

The cooling rate was calculated as

|  | $CR= \frac{\Delta T}{t_{total}}\cdot60 (℃/min)$ | S25 |
| --- | --- | --- |

where Δ*T* is the temperature zone from −20 °C to −140 °C, which is generally the most relevant zone for vitrification and effective rewarming. All parameters used for the physical properties of cryomesh and CPA are listed in Table S5.

Threshold of cooling and rewarming rate. The 7.5 × 10⁴ °C min⁻¹ cooling line and 1.4 × 10⁵ °C min⁻¹ rewarming line are retained as protocol-specific empirical benchmarks because *F. fungites* larvae show high viability, even in limited trials. Neither rate was measured directly in larvae. Because larval availability was limited, cooling was measured using CPA-loaded 100-µm polyethylene (PE) particles (Cospheric LLC) as physical replicas, and the particle response was used to estimate larval cooling. Rewarming was estimated using the same protocol with the heat transfer model. These benchmarks were not derived from a direct coral measurement experiment. Meeting the benchmarks indicates thermal compatibility with prior success; accordingly, the model is presented as necessary but not sufficient for predicting recovery.

Predictive scope. The inverse volume-rate relationship is a physical trend within the model, but volume alone does not determine post-rewarming recovery. The *P. meandrina* results and mechanically sheared *D. cylindrus* provide explicit counterexamples to a volume-only interpretation. Favorable predicted rates do not remove limitations for species and CPA. Cryoprotectant loading and toxicity, osmotic injury, membrane permeability, developmental stage, morphology, and mechanical damage can all alter larval viability.

**Table S5. Detailed physical properties for heat transfer simulation.**

|  | **Parameters** | **Value** |
| --- | --- | --- |
| Cryomesh property | Density – Stainless steel (ρ_m_) | 8030 kg/m³ |
|  | Specific heat capacity (c_p,m_) | 502 J/kg/K |
|  | Thermal conductivity (k_m_) | 12.1 W/m/K |
|  | Mesh wire diameter (*D*) | 30 µm |
|  | Mesh pore size (*P*) | 30-35 µm |
|  | Solid fraction (*Φ*) | 0.5 |
| Cryoprotectant property | Density (ρ_b_) | 1,101.4 kg/m³ |
|  | Specific heat capacity (c_p,b_) | 1,400 J/kg/K |
|  | Thermal conductivity (k_b_) | 1.6 W/m/K |

For the modeling, we used simplified thermal properties and conditions. The model didn’t consider latent heat, crystallization/devitrification kinetics, temperature-dependent properties, or nonuniform/partial mesh contact. The convection heat transfer coefficient for cooling is h = hc = 1,250 W/m²/K. The convection heat transfer coefficient for rewarming is h = hc = 2,500 W/m²/K at both 26 and 50 °C. Coefficients are assumed constant in time and uniform over each exposed area. We used those values to fit the experimental cooling and rewarming rate. The heat transfer coefficient could change due to vapor layer presenting, CPA remaining, or slow plunging speed.

**Figure S8. Theoretical cooling rate of coral larvae.** **A)** Theoretical cooling rate as a function of larval volume across all tested coral species and development stages, showing an inverse relationship between larval size and cooling rate. **B)** Coral larvae of different species and development stages can be vitrified as the theoretical cooling rate passes the estimated threshold cooling rate based on Fungia fungites cryopreservation experiments (Guo et al. 2024).

**Figure S9. Theoretical rewarming rates of coral larvae at different developmental stages.** **A, B)** *Lobactis scutaria* and **C, D)** *Pocillopora meandrina* under ambient rewarming temperatures of 24-26 °C (left) and 50 °C (right). Boxplots represent the distribution of theoretical rewarming rates for each developmental stage, with black markers indicating individual simulations based on measured larvae volume. “Sheared” groups represent mechanically processed larvae. The dashed red line is the estimated critical rewarming rate required to minimize devitrification during vitrification-based cryopreservation.

**Figure S10. Theoretical rewarming rates of coral larvae at different developmental stages** **A, B)** *Colpophyllia natans*, **C, D)** *Dendrogyra cylindrus*, and **E, F)** *Pseudodiploria strigosa* and under ambient rewarming temperatures of 24-26 °C (left) and 50 °C (right). Boxplots represent the distribution of theoretical rewarming rates for each developmental stage, with black markers indicating individual simulations based on measured larvae volume. “Sheared” groups represent mechanically processed larvae. The dashed red line is the estimated critical rewarming rate required to minimize devitrification during vitrification-based cryopreservation.

**Figure S11. Theoretical rewarming rates of coral larvae at different developmental stages.** **A, B)** *Mussa angulosa* and **C, D)** other species under ambient rewarming temperatures of 24-26 °C (left) and 50 °C (right). Boxplots represent the distribution of theoretical rewarming rates for each developmental stage, with black markers indicating individual simulations based on measured larvae volume. “Sheared” groups represent mechanically processed larvae. The dashed red line is the estimated critical rewarming rate required to minimize devitrification during vitrification-based cryopreservation.

**Macro for processing larval volumes in ImageJ**

***Set scale with micrometer***

- Keep unit of length in pixels (pixels per 1 mm)
- Known distance: keep blank
- Set Measurements: fit ellipse

***Establish threshold for a set of images and record macro***

- Open one of the images from a set of images
- Click “Plugins”, “Macros”, and “Record” – brings up the macro recorder window
- Click “Image”, “Type”, “8-bit” – turns image grayscale
- Click “Image”, “Adust”, and “Threshold” – the Threshold window will open
  - Adjust the top sliding scale until the larvae or egg in image is represented in red
  - Record this value (e.g., 75, 255)
  - This gives an approximate threshold for the sample images
- Click “Create” button in the macro recorder window
  - The “Macro.ijm” window will open
  - Keep this open while processing images
  - Copy and paste one of the following codes into that window

**For brightfield images, use:**

run("8-bit");

setAutoThreshold("Default");

run("Threshold...");

setThreshold(0, 255);

run("Convert to Mask");

run("Fill Holes");

**For darkfield images use:**

run("8-bit");

setAutoThreshold("Default");

run("Threshold...");

setThreshold(75, 255);

setOption("BlackBackground", true);

run("Convert to Mask");

run("Fill Holes")

- The image sequence will appear in one window; scroll through them to check quality
- Select the “Macro.ijm” window and click, “Run”
  - This will process all the images in the sequence with the macro code.
  - Select “OK” for several processes (e.g., “Convert Stack to Binary”, click “OK”)

***Measurements can now be obtained for images in the sequence***

- To select the egg/larva, use the “Wand (tracing) tool”
- Once specimen is outlined in yellow, click “CMD+M” (Mac) or “CTRL+M” (PC) to get measurements
  - Depending on quality of images, it’s possible to use this process for all images
  - However, if larvae or eggs are along an edge or touch another egg/larva, those images will need to be traced by hand with the “polygon tool”
